# Hematological parameters and major adverse cardiovascular events: a prospective study in a Chinese population involving 2,970 participants

**DOI:** 10.7150/ijms.104118

**Published:** 2025-03-24

**Authors:** Hongna Mu, Xinyue Wang, Xianghui Zhao, Ruiyue Yang, Wenduo Zhang, Hongxia Li, Siming Wang, Fusui Ji, Wenxiang Chen, Jun Dong, Xue Yu

**Affiliations:** 1 The Key Laboratory of Geriatrics, Beijing Institute of Geriatrics, Institute of Geriatric Medicine, Chinese Academy of Medical Sciences, Beijing Hospital/National Center of Gerontology of National Health Commission, P.R. China.; 2 Department of Cardiology, Beijing Hospital, National Center of Gerontology; Institute of Geriatric Medicine, Chinese Academy of Medical Sciences, P. R. China.; 3 National Center for Clinical Laboratories, Institute of Geriatric Medicine, Chinese Academy of Medical Sciences, Beijing Hospital/National Center of Gerontology, P.R. China.

**Keywords:** Coronary artery disease, Major adverse cardiovascular events, Hematologic parameters

## Abstract

Hematological parameters are among the most accessible and routinely performed clinical tests. Recent studies have gradually revealed their potential for risk prediction. This study aimed to assess the association between hematological parameters and major adverse cardiovascular events (MACEs) in patients with coronary artery disease. This prospective study included 2,970 Chinese participants who underwent coronary angiography, with hematological and biochemical indicators measured at baseline. MACEs, comprising myocardial infarction, stroke, revascularization, and all-cause mortality, were recorded during follow-up. Univariate and multivariate analyses were conducted to evaluate the relationship between the hematological parameters and MACEs. Over a median follow-up period of 79 months, 474 MACEs were documented. Kaplan-Meier analysis indicated that the participants with lower levels of RBC, PLT, PCT, LYMPH% and BASO%, as well as higher RDW-CV, RDW-SD, MONO% and NEUT%, exhibited reduced survival probability. Multivariate Cox regression analysis identified elevated RDW-CV as a significant risk factor for MACE (T3 HR, 1.292; 95% CI, 1.013-1.647; *P*=0.039), while lower BASO% demonstrated a protective effect (T3 HR, 0.750, 95 % CI: 0.591-0.953; *P*=0.018). LYMPH% also showed a significant association with MACEs. Additionally, nonlinear correlations were observed between PLT and PCT and MACEs. In conclusion, RDW-CV, BASO%, PLT, PCT and LYMPH% were closely associated with MACEs and may serve as potential predictors for cardiovascular risk in patients with coronary artery disease.

## 1. Background

Coronary artery disease (CAD) has become the leading cause of mortality worldwide imposing an increasingly significant burden on global populations. The primary pathological mechanism underlying CAD is atherosclerosis, a process that is difficult to reverse. Therefore, at the early stages of the disease, it is crucial to identify additional modifiable risk factors for atherosclerosis that can facilitate early diagnosis and prompt intervention 1.

Hematological parameters are among the most readily available and accessible clinical tests in routine practice. In recent years, a growing body of evidence has emphasized the importance of these parameters in predicting outcomes for patients with CAD 2, 3. For example, red cell distribution width (RDW), commonly expressed as RDW-standard deviation (RDW-SD) and RDW-coefficient of variation (RDW-CV), reflects the variation in red blood cell (RBC) size. Elevated RDW has been associated with atherosclerosis progression 4, CAD severity 5, and increased mortality and cardiovascular events 6, 7. In addition to RDW, elevated neutrophil counts have been linked to CAD, supporting the notion that high neutrophil levels are a causal risk factor for the disease 8. Furthermore, platelet counts have been found to correlate with mortality and future cardiovascular disease risk in the general population 9. Given the widespread availability and ease of measurement of hematological parameters in clinical settings, they present significant potential as predictive markers for cardiovascular events. This study aims to explore the unique role of hematological parameters, particularly RDW-SD, RDW-CV, and their associations with major adverse cardiovascular events (MACEs), in a cohort of Chinese patients.

## 2. Methods

### 2.1 Patient selection

This prospective cohort study was conducted at Beijing Hospital. Between 2017 and 2020, a total of 2970 patients undergoing coronary angiography were enrolled after providing written informed consent 10. This study protocol was approved by the Ethics Committee of Beijing Hospital (2016BJYYEC-121-06) and was registered at ClinicalTrials.gov (Identifier: NCT03072797, Dated: March 7, 2017). Patients were excluded if they had any of the following criteria: severe congenital heart disease, severe cardiac insufficiency, primary pulmonary hypertension, hepatic or renal dysfunction, and severe peripheral arterial disease or any conditions contraindicating cardiac catheterization. Additionally, patients who had undergone radiotherapy or chemotherapy, pregnant or breastfeeding women, individuals with a history of alcoholism or drug abuse, and those receiving treatment for mental illness were excluded from the study. At baseline, data on all patients' demographic, medical history, smoking habits, and body mass index (BMI) were collected. Intravenous blood samples were taken before the angiographic procedure, and serum was separated, aliquoted and stored at -80°C for subsequent analysis.

### 2.2 Follow-up time and study endpoint

The study endpoint was defined as a composite of MACEs, which included myocardial infarction (MI), stroke, revascularization, or all-cause mortality. All-cause mortality included deaths due to cardiac, vascular and non-cardiovascular causes. Patients were monitored annually, with adverse events recorded at each follow-up visit. For analysis, only the first occurrence of an adverse event was considered. The primary methods of follow-up were telephone interviews and the review of outpatient medical records. The final follow-up date was January 17, 2024, and a total of 334 participants were lost to follow-up.

### 2.3 Hematological parameters assays

Hematological parameters were analyzed using an automated analyzer (Sysmex, Kobe, Japan). The parameters included RBC and associated measurements such as hematocrit (HCT), mean corpuscular volume (MCV), mean corpuscular hemoglobin (MCH), mean corpuscular-hemoglobin concentration (MCHC), RDW-CV and RDW-SD. Platelet (PLT) count and related factors, including plateletcrit (PCT), platelet distribution width (PDW), mean platelet volume (MPV), and platelet-large cell ratio (P-LCR), were also assessed. In addition, white blood cell (WBC) count and its subpopulations—neutrophils (#NEUT), lymphocytes (#LYMPH), monocytes (#MONO), basophils (#BASO), eosinophils (#EOS)—as well as the percentage of each type (NEUT%, LYMPH%, MONO%, EOS%, BASO%) were measured. Hemoglobin (HGB) levels were also included in the analysis.

### 2.4 Other parameters and laboratory testing

Serum concentrations of fasting blood glucose (FBG), total cholesterol (TC), triglycerides (TG), high density lipoprotein cholesterol (HDL-C), low density lipoprotein cholesterol (LDL-C), creatinine (Crea) and uric acid (UA) were measured using commercial kits (Sekisui Medical Technologies, Osaka, Japan) on a 7180 chemistry analyzer (Hitachi, Tokyo, Japan). To ensure accuracy and consistency, two quality control materials were prepared by mixing fresh serum samples. These controls were analyzed alongside the patient samples in each assay run, including both the liquid chromatography-tandem mass spectrometry method and the laboratory assays, to monitor the performance and reliability of the measurements.

### 2.5 Statistical analysis

All variables were assessed for normality. Normally distributed parameters were expressed as mean ± standard deviation (SD), while skewed variables were presented as median and interquartile range (IQR) (25th to 75th percentile). Count data was reported as frequencies and percentages. Differences in continuous variables were evaluated using the T-test or analysis of variance (ANOVA) for normally distributed data, while the nonparametric Jonckheere-Terpstra test was used for variables with non-normal distributions. Categorical variables were compared using the Chi-square test. Kaplan-Meier analysis was performed to calculate the survival rate, stratified by tertiles of hematological parameters, and survival curves were compared using the log-rank test. Univariate and multifactorial Cox proportional hazard models were applied to assess the association between hematological parameters and MACEs. In Model 2, adjustments were made for age and gender. In Model 3, further adjustments were performed for age, gender, obesity, smoking status, hypertension, dyslipidemia, diabetes, history of stroke, and family history of premature CAD. Additionally, restricted cubic spline (RCS) regression was employed to detect potential nonlinear relationships between PLT, PCT, and the prevalence of MACEs. All statistical analyses were performed using IBM SPSS Statistics v. 25.0 software (IBM Corp., Armonk, NY, USA).

## 3. Results

A total of 2,970 participants were included at the beginning of the study, with 334 participants lost to follow-up. During a median follow-up period of 79 (65, 93) months, 474 MACEs were recorded. These events included 49 myocardial infarctions, 81 strokes, 220 revascularizations, and 208 all-cause deaths (91 cardiovascular deaths and 117 non-cardiovascular deaths). Additionally, there were cases of combined events, including:

- 2 cases of MI complicated with both stroke and revascularization,
- 5 cases of MI complicated with revascularization and all-cause death,
- 1 cases of MI complicated with stroke,
- 33 cases of MI complicated with revascularization,
- 4 cases of MI complicated with all-cause death,
- 10 cases of stroke complicated with revascularization,
- 7 cases of stroke complicated with all-cause death,
- 26 cases of revascularization complicated with all-cause death.

Table 1 presents the baseline characteristics of the study population stratified by the occurrence of MACE. As expected, older participants had a higher prevalence of MACE, and a greater proportion of men experienced MACE compared to women. The MACE group had significantly lower BMI, diastolic blood pressure (DBP), and HDL-C, but higher systolic blood pressure (SBP) and Crea levels compared with the non-MACE group. Participants in the MACE group were also more likely to have a history of hypertension, diabetes, and stroke.

In addition, the MACE group exhibited higher RDW-CV, #NEUT, #MONO, NEUT% and MONO%, but lower HCT, PLT, PCT, #LYMPH, #BASO, LYMPH% and BASO%, compared to the non-MACE group. There were no significant differences between the two groups in terms of current smoking status, overweight/obesity, dyslipidemia, family history of premature CAD, statins use, FBG, TC, TG, LDL-C, UA, or other hematologic indices.

**Figure 1:**
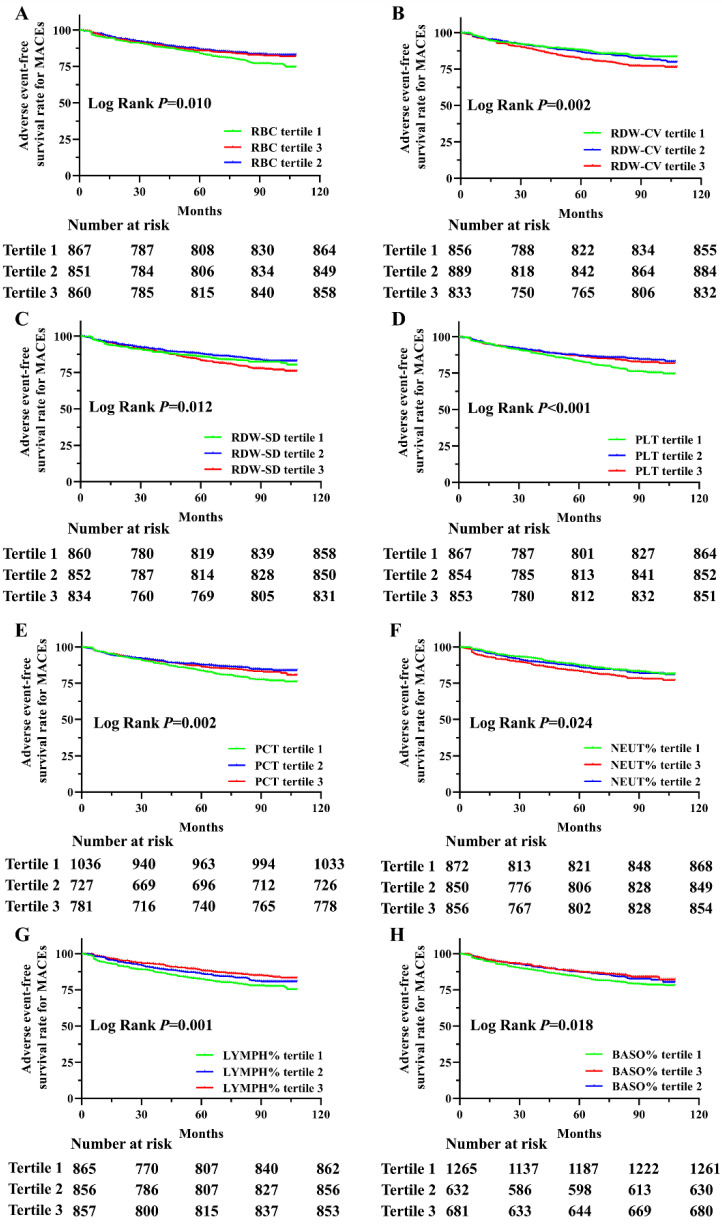
** Kaplan-Meier survival analysis of adverse event-free survival rate for MACEs based on RBC, RDW-SD, RDW-CV, PLT, PCT, NEUT%, LYMPH% and BASO% in this population.** Adverse event-free survival rate for MACEs in this population divided by tertiles of RBC (A), RDW-CV (B), RDW-SD (C), PLT (D), PCT (E), NEUT% (F), LYMPH% (G) and BASO% (H) levels during the follow-up period.

Next, we analyzed the adverse event-free survival rate for MACEs, MI, stroke, revascularization, all-cause deaths, cardiovascular deaths and non-cardiovascular deaths in this cohort, divided by tertiles of hematologic indices during the follow-up period (Supplement table 1). Significant survival differences were observed among the tertiles of RBC, RDW-CV, RDW-SD, PLT, PCT, NEUT%, LYMPH% and BASO% in relation to MACEs, as shown in Figure 1. The analysis revealed that the participants with lower RBC, PLT, PCT, LYMPH% and BASO%, as well as higher RDW-CV, RDW-SD and NEUT%, had reduced survival probabilities. In addition, MONO% was also significantly associated with MACEs (*P*=0.040, Supplement Figure 1). Although adverse event-free survival rates for MI and stroke were not statistically significant for any hematological indicators, multiple indicators showed significant associations with revascularization, all-cause deaths, cardiovascular deaths and non-cardiovascular deaths. Adverse event-free survival rates differed significantly for all-cause, cardiovascular, and non-cardiovascular deaths. Significant associations with tertiles of RBC, HGB, RDW-CV, RDW-SD, PLT, #LYMPH, NEUT%, and LYMPH% were observed across all outcomes. Additionally, cardiovascular death survival differences were significant for MPV, PDW, P-LCR, #MONO, and MONO%, while non-cardiovascular deaths were associated with HCT and PCT (Supplement table 1).

**Figure 2:**
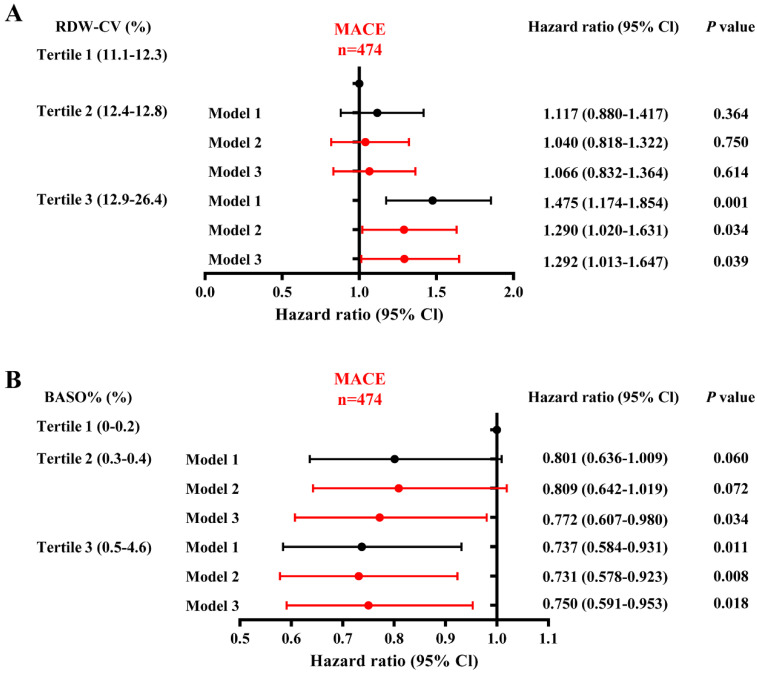
** Forest plots of univariate and multivariate Cox subgroup analysis of RDW-CV and BASO% on the incidence of MACEs in this population.** Model 1: Crude risk. Model 2: Adjusted for age and gender. Model 3: Further adjusted for smoking status, obesity or overweight, hypertension, dyslipidemia, diabetes, stroke and family history of premature CAD. *p* < 0.05 considered statistically significant.

Univariate and multivariate Cox proportional hazard models were used to further evaluate the associations between hematologic indices and MACEs, MI, stroke, revascularization, all-cause deaths, cardiovascular deaths and non-cardiovascular deaths (Supplement table 2-8). In univariate Cox proportional hazard models, participants in the highest tertile of RDW-CV levels had a significantly increased risk of MACEs compared to those in the lowest tertile (T3 HR, 1.475; 95% CI, 1.174-1.854; *P*=0.001). After adjusting for age, gender, obesity, smoking status, hypertension, dyslipidemia, diabetes, history of stroke and family history of premature CAD, elevated RDW-CV levels remained a significant risk factor for MACEs (T3 HR, 1.292; 95% CI, 1.013-1.647; *P*=0.039). Additionally, BASO% demonstrated a significant protective effect against incident MACEs (T3 HR, 0.737; 95% CI, 0.584-0.931; *P*=0.011) in Model 1. After adjusting for age and gender, the HR decreased to 0.731 (95 % CI, 0.578-0.923; *P*=0.008) and increased slightly to 0.750 (95 % CI: 0.591-0.953; *P*=0.018) after further adjustments for additional covariates (Supplement table 2 and Figure 2).

Univariate Cox analysis indicated that RBC, HCT, PLT, and PCT were significant protective factors against MACEs, while PDW, NEUT% and MONO% were risk factor for MACEs (all *P*≤0.05). In the multivariate Cox analysis, LYMPH% also showed a correlation with MACEs (*P*=0.042), with the highest tertile (T3) and middle tertile (T2) showed completely opposite associations in Model 2 and 3 (Supplement table 2).

None of the hematologic indices were associated with MI or stroke (Supplement table 3-4). RDW-SD, LYMPH% and BASO% demonstrated significant associations with revascularization, both before and after adjustment. MCV, WBC and #NEUT were associated with revascularization in the univariate Cox analysis, while MCV and MCH maintained significant relationships with revascularization in Model 2 (Supplement table 5).

High levels of RDW-CV, RDW-SD, MPV, P-LCR, and NEUT% were associated with higher all-cause mortality, whereas low PLT and LYMPH% was linked to increased all-cause mortality in the fully adjusted model. Interestingly, PCT tertiles (T2 and T3) exhibited different relationships with all-cause mortality. HGB, HCT, MCHC, #LYMPH, #MONO and MONO% reached statistical significance only in univariate Cox analysis, while RBC and PDW were associated with all-cause mortality in Model 1 and 2 (Supplement table 6).

For non-cardiovascular death, only RDW-CV consistently showed associations across all three models. HGB, HCT, PLT, PCT, #LYMPH, NEUT%, LYMPH% and MONO% were associated with non-cardiovascular death without adjustment. RBC showed the associations in Model 1 and 2 (Supplement table 7).

For cardiovascular death, several indicators including PLT, MPV, PDW, P-LCR, #NEUT, NEUT%, LYMPH% and MONO% showed significant association. RBC, RDW-SD, PCT and #LYMPH were associated with cardiovascular death in univariate Cox analysis. And RDW-CV remained significantly associated with cardiovascular death in Model 1 and 2 (Supplement table 8).

**Figure 3:**
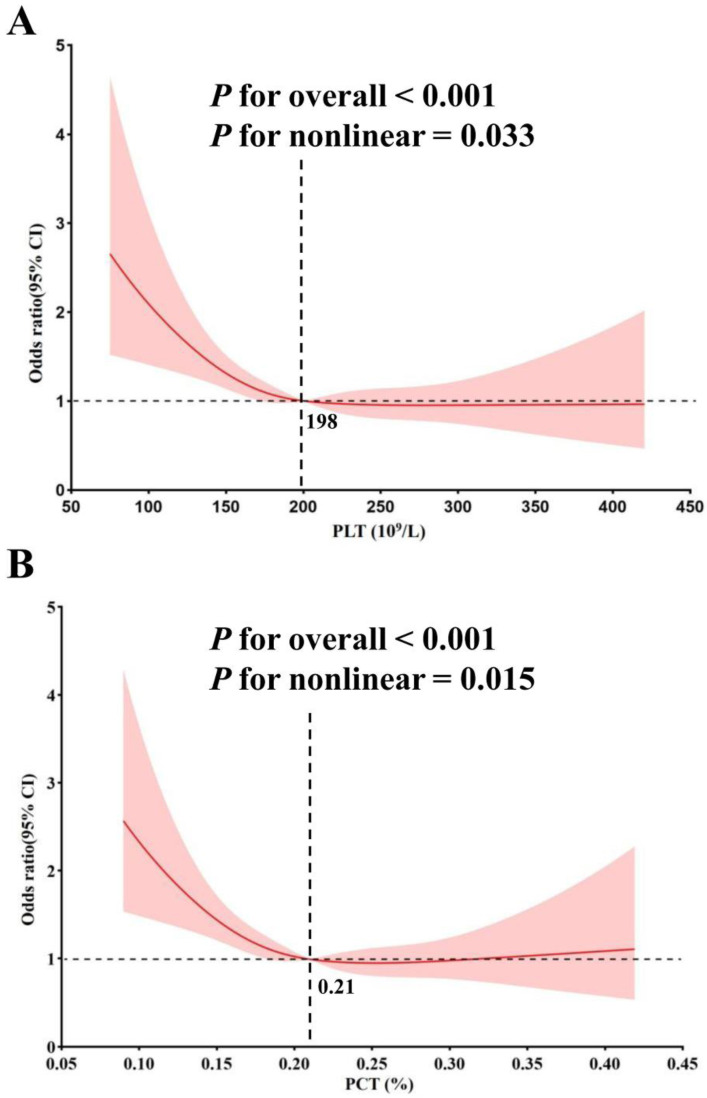
** Non-linear association between PLT, PCT and MACEs by the restricted cubic spline model.** The turning point for PLT (A) is 198 (10^9^/L) and the turning point for PCT (B) is 0.21%.

Given the completely opposite associations observed in the highest tertile (T3) and middle tertile (T2) of PLT and PCT with MACEs, we conducted a nonlinear correlation analysis. The RCS regression suggested a statistically significant nonlinear correlation between PLT and PCT levels and MACEs. Initially, there was a rapid decline in MACEs risk as PLT and PCT levels increased. However, the incidence rate of MACEs plateaued once PLT surpassed 198, while beyond certain thresholds (PCT > 0.21), the incidence of MACEs began to increase gradually (Figure 3).

**Figure 4:**
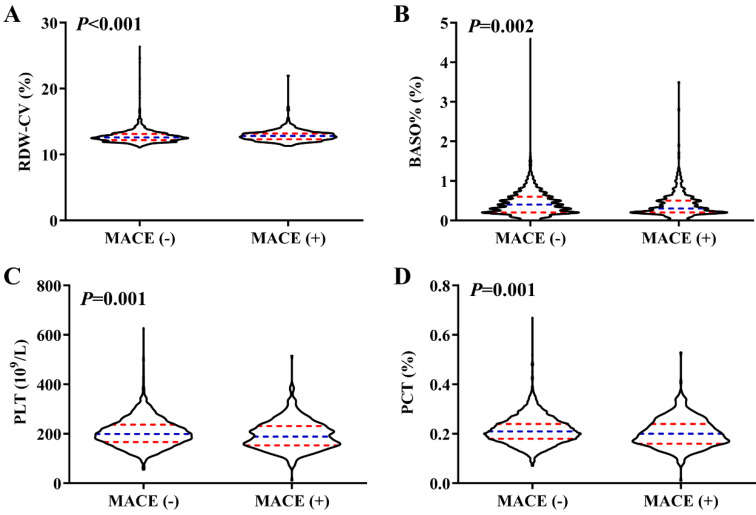
** The relations of levels of RDW-CV, BASO%, PLT and PCT with MACE.** Violin plots of RDW-CV (A), BASO% (B), PLT (C) and PCT (D) at presentation with MACE (-) and MACE (+). *p* < 0.05 considered statistically significant.

**Figure 5:**
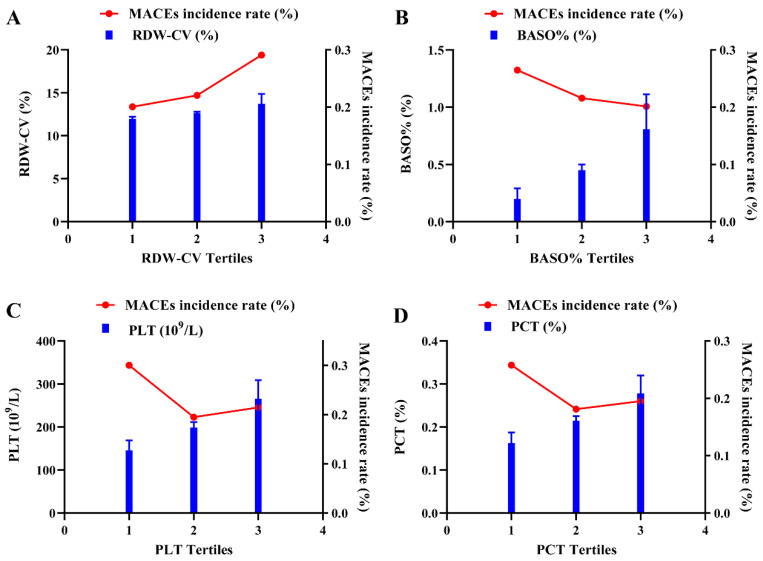
** The association of tertiles of RDW-CV, BASO%, PLT and PCT and MACEs incidence rate.** The double ordinate plots show the association of tertiles of RDW-CV (A), BASO% (B), PLT (C) and PCT (D) and MACEs incidence rate.

Detailed results for RDW-CV, BASO%, PLT and PCT in different groups are presented in Figure 4. Overall, participants in the MACE group exhibited higher RDW-CV and lower PLT, PCT and BASO%. Consistently, the incidence of MACEs increased with higher RDW-CV levels, whereas BASO% showed an inverse trend. For PLT and PCT, participants in the T1 group had the highest risk for composite cardiovascular events, while those in the T2 group had the lowest MACEs incidence rate. The T3 group exhibited a higher incidence than T2 but lower than T1, which reflected the observed nonlinear relationship between PLT, PCT and MACEs (Figure 5).

## 4. Discussion

In this study, we investigated the association between hematological parameters and MACEs in a Chinese prospective cohort undergoing coronary angiography. These readily available parameters demonstrated significant value in cardiovascular risk estimation. Our primary findings revealed that higher RDW-CV levels and lower BASO% were associated with an increased risk of cardiovascular events. Additionally, a nonlinear relationship between PLT, PCT, and MACEs was identified, and LYMPH% also showed a significant association with MACEs.

RBCs, also known as erythrocyte, are non-nucleated blood cells with a characteristic biconcave shape, primarily responsible for oxygen and carbon dioxide transport. Beyond this traditional role, RBCs perform diverse physiological functions 11. RDW, expressed as either RDW-SD or RDW-CV, reflects the variation in RBC size and has been widely used to diagnose hematological disorders 12. In recent years, RDW has emerged as a potential risk factor for CAD, and has been associated with poor outcomes in stroke 13, MI 14, percutaneous coronary intervention 15, heart failure 16 and secondary cardiovascular adverse events 2, 17. However, these studies often did not differentiate between RDW-SD and RDW-CV, conflicting evidence exists regarding RDW-SD and RDW-CV. One study suggested RDW-SD as a better predictor of severe coronary artery calcification 12, while another study highlighted that RDW-CV could predict cardiovascular outcomes in patients with extensive aortoiliac disease 18. Our findings confirmed that elevated RDW-CV was associated with an increased risk of MACEs. The precise mechanisms linking increased RDW-CV to worse outcomes remain unclear. Prior studies have shown that RDW-CV correlated with impaired erythrocyte deformability 19 and elevated inflammatory markers 20, both of which play critical roles in CAD progression 21.

Basophils, the least abundant leukocyte subtype in human peripheral blood 22, remain underexplored in the context of CAD. Functionally similar to mast cells, basophils play dual roles in IgE-mediated inflammation, exhibiting both pro- and anti-inflammatory effects 23. A recent case-control study found reduced BASO% levels in patients with early-onset CAD and myocardial infarction, with significant correlations to disease severity 24. In our study, we found lower BASO% in the MACE group, with a clear trend showing decreased MACEs risk as BASO% levels increased. However, the precise role of basophils in CAD and their association with cardiovascular outcomes remain unclear and warrant further investigation.

PLTs are small, anucleated blood cells, continuously produced from megakaryocytes 25. Beyond their well-known role in vascular hemostasis, PLTs have garnered attention for their involvement in multiple physiological and pathophysiological processes, including inflammation, autoimmunity, and the initiation and progression of atherosclerosis 26. Activated PLTs adhere to damaged vessel walls or activated endothelium, facilitating the recruitment of leukocytes and the formation of leukocyte-platelet aggregates, which are critical for the development of atherosclerotic lesions 27. PLT function has been linked to acute MI, arterial and venous thrombosis, and strokes 28. Moreover, PLTs have been identified as an independent risk factor for cardiovascular disease, with a U-shaped relationship between PLT count and mortality in both elderly and general populations 9, 29. Similar to these findings, our study identified a nonlinear relationship between PLTs and MACEs. While prior research has largely overlooked nonlinear correlations, we observed that, the MACE group had lower overall PLT levels (Table 1 and Figure 4). Specifically, as PLT levels increased, the risk of MACEs decreased rapidly until reaching a threshold of 198 (10⁹/L), beyond which the risk flattened. This turning point differs from previous studies, likely due to differences in the study population. Increased platelet mass may reflect heightened platelet activity, potentially leading to a destructive inflammatory response and a prothrombotic state 30. Conversely, lower PLT counts increased the risk of MACEs, it may be due to the overlooked protective functions of PLTs, including immune regulation, cellular migration, proliferation, and angiogenesis, all of which are essential for tissue healing 31. These findings highlight the need for further research on PLT function, particularly through large-scale, prospective cohort studies.

PCT, defined as the percentage of blood volume occupied by PLTs, serves as an indicator of evaluate platelet activity 30, 32. PCT is the product of the platelet counts and MPV, providing a more comprehensive measure of total platelet mass. Previous studies have demonstrated that higher PCT levels correlate with worse cardiovascular outcomes in CAD patients 33. In contrast, our study observed reduced PCT levels in patients with MACEs (Table 1 and Figure 4), along with a nonlinear relationship between PCT and MACEs (Figure 3). Initially, the risk of MACEs decreased rapidly as PCT increased, but beyond a threshold of 0.21, the risk began to rise slowly with further increases in PCT. The discrepancy between our findings and previous studies may stem from the following factors. First, earlier studies often failed to account for nonlinear relationships and overlooked the protective functions of PLTs. Second, differences in study populations may also play a role. Our cohort consisted of patients undergoing coronary angiography, a population with elevated CAD risk and significant cardiovascular risk factors. Further studies are necessary to clarify the role of PCT in predicting CAD risk in diverse populations.

There are other findings about this paper. Obesity, typically defined as BMI ≥ 25 kg/m², is widely recognized as a risk factor for CAD 34, though its impact on CAD and mortality in the elderly remains controversial 35. BMI often demonstrates a U- or J-shaped relationship with clinical outcomes and mortality 36, a phenomenon referred to as the 'obesity paradox,' which may be largely attributed to differences in lean vs fat body mass 35. In our study, with an average participant age of 65.6 years and a BMI of 25.7, the MACE group had significantly lower BMI 10, suggesting that BMI may be an imperfect indicator of obesity, especially in older adults. Similarly, while lowering blood pressure reduces cardiovascular risk, a U-shaped relationship between baseline DBP and CAD events has also been reported 37, 38. Our study observed lower baseline DBP in the MACE group, suggesting that low DBP may contribute to myocardial damage and CAD events. Finally, as expected, the MACE group showed significantly lower HDL-C levels, consistent with its established role as a protective factor for CAD 39 (Table 1).

During a median follow-up period of 79 (65, 93) months, our study documented 208 all-cause deaths, including 91 cardiovascular deaths and 117 non-cardiovascular deaths. Notably, the adverse event-free survival rates for all-cause deaths, cardiovascular deaths, and non-cardiovascular deaths were not identical (Supplementary table 1). In this study, the cause of non-cardiovascular deaths were mainly cancer, severe pneumonia and organ failure, influenced overall survival analyses and may have indirectly contributed to cardiovascular events 40-44.

Stroke is a critical component of cardiovascular risk, and promoting stroke recovery has been shown to improve cardiovascular function 45. During the follow-up, 81 strokes were recorded, but no hematological parameters showed significant associations with stroke incidence. This finding may be due to the limited follow-up time and sample size, further studies are needed to elucidate these relationships (Supplementary table 1 and 4).

In addition, although chronic inflammatory disorders (such as lupus or rheumatoid arthritis), thalassemia, primary bone marrow disorders, recent blood transfusions, vitamin B12 deficiency, or folate deficiency were not specifically excluded in this population, there were no patients with these conditions. There were no patients with severe anemia; however, we did not exclude those with mild to moderate anemia. In this population, 21 individuals had hemoglobin levels less than 90 g/L, and 534 individuals had hemoglobin levels less than 120 g/L.

This study has several limitations. Firstly, as a single-center study, the generalizability of the results is limited, and further multicenter studies are needed to validate the results. Secondly, although the patients recruitment was completed by June 2019, prior to the outbreak of COVID-19, the follow-up period coincided with the COVID-19 pandemic, which may have contributed to an increased incidence of MACEs. Additionally, the participants in our study were either suspected of having CAD or had a history of CAD and underwent coronary angiography, meaning they were not from a truly healthy population. The control group already had high levels of cardiovascular risk factors, potentially leading to an underestimation of the associations between hematological parameters and CAD. Lastly, blood samples were only collected at baseline during hospitalization, and no follow-up samples were taken. While baseline data was used to assess the predictive ability of hematological parameters, the lack of repeated blood measurements may limit the robustness of our conclusions. Future studies should include follow-up blood sampling to provide a more accurate assessment of the long-term predictive value of hematological parameters for cardiovascular events.

## 4. Conclusions

The major findings of the present study are as follows: MACEs were more prevalent in patients with high RDW-CV and low BASO%. And a nonlinear relationship between PLT, PCT, and MACEs was identified. Additionally, LYMPH% showing a significant correlation with cardiovascular outcomes.

## Supplementary Material

Supplementary figure and tables.

## Figures and Tables

**Table 1:** Comparison of baseline characteristics of study population according to the occurrence of MACEs.

Characteristic^ a^	MACE(-)	MACE (+)	*P*
n	2162	474	-
Age, y	64.96±10.74	68.25±11.36	<0.001
Male, n (%)	1344(62.16)	325(68.57)	0.011
BMI, kg/m2	25.79±3.53	25.26±3.41	0.003
SBP, mmHg	136.36±17.83	138.51±20.58	0.043
DBP, mmHg	78.68±11.31	77.12±11.07	0.029
Current smoker, n (%)	680(31.45)	165(34.81)	0.299
Overweight/obesity, n (%)	1501(69.43)	301(63.50)	0.131
Hypertension, n (%)	1436(66.42)	350(73.84)	0.001
Diabetes, n (%)	730(33.77)	201(42.41)	<0.001
Dyslipidemia, n (%)	989(45.74)	206(43.46)	0.359
History of stroke, n (%)	207(9.57)	75(15.82)	<0.001
Family history of premature CAD, n (%)	174(8.05)	27(5.70)	0.120
**Statins use, n (%)**			0.350
No	1198(55.41)	257(54.22)	
Take statins intermittently	204(9.44)	30(6.33)	
Take statins continuously Over 1 year, n (%)	557(25.76)	135(28.48)	
FBG, mmol/L	6.64±2.22	6.90±2.34	0.107
TC, mmol/L	3.91±0.93	3.92±0.96	0.324
TG, mmol/L	1.27 (0.90~1.75)	1.31 (0.94~1.81)	0.220
HDL-C, mmol/L	1.04 (0.89~1.21)	0.99 (0.85~1.17)	<0.001
LDL-C, mmol/L	2.25 (1.77~2.82)	2.29 (1.79~2.86)	0.364
Crea, μmol/L	71.01±16.86	75.09±18.92	<0.001
UA, μmol/L	326.18±85.43	332.35±97.66	0.107
RBC, 10^12^/L	4.32±0.50	4.25±0.57	0.145
HGB, g/L	132.00 (122.00~143.00)	130.00 (119.25~142.00)	0.064
HCT, %	38.40 (35.70~41.20)	37.70 (34.90~41.18)	0.030
MCV, fL	89.20 (86.70~91.70)	88.90 (86.40~92.00)	0.861
MCH, pg	30.70 (29.70~31.60)	30.60 (29.50~31.70)	0.735
MCHC, g/L	343.00 (337.00~351.00)	343.00 (336.25~352.00)	0.931
RDW-CV, %	12.60 (12.20~13.10)	12.80 (12.30~13.20)	<0.001
RDW-SD, fL	40.70 (39.00~42.50)	40.90 (39.10~43.10)	0.052
PLT, 10^9^/L	199.00 (166.00~237.00)	188.00 (153.00-~231.00)	0.001
PCT, %	0.21 (0.18~0.24)	0.20 (0.16~0.24)	0.001
MPV, fL	10.50 (9.90~11.10)	10.50 (9.90~11.20)	0.203
PDW, %	12.20 (11.00~13.50)	12.30 (11.10~13.78)	0.114
P-LCR, %	28.70 (23.90~34.00)	29.40 (24.40~35.30)	0.134
WBC, 10^9^/L	6.11 (5.25~7.24)	6.19 (5.19~7.48)	0.222
#NEUT, 10^9^/L	3.66 (2.90~4.57)	3.80 (3.06~4.73)	0.035
#LYMPH, 10^9^/L	1.74 (1.38~2.20)	1.68 (1.28~2.15)	0.032
#MONO, 10^9^/L	0.44 (0.35~0.54)	0.45 (0.36~0.58)	0.016
#BASO, 10^9^/L	0.02 (0.01~0.04)	0.02 (0.01~0.03)	0.025
#EOS, 10^9^/L	0.12 (0.07~0.19)	0.13 (0.07~0.19)	0.593
NEUT%, %	60.00 (53.90~66.50)	61.20 (55.10~67.80)	0.011
LYMPH%, %	29.40 (23.50~35.30)	27.50 (21.73~33.60)	<0.001
MONO%, %	7.20 (6.00~8.60)	7.30 (6.10~8.90)	0.039
BASO%, %	0.40 (0.20~0.60)	0.30 (0.20~0.50)	0.002
EOS%, %	2.00 (1.20~3.10)	1.90 (1.20~3.20)	0.849

Not all data were available for all patients. Abbreviations: MACE, major adverse cardiovascular event; BMI, body mass index; SBP, Systolic blood pressure; DBP, Diastolic blood pressure; FBG, fasting blood glucose; TC, total cholesterol; TG, triglyceride; HDL-C, high density lipoprotein cholesterol; LDL-C, low density lipoprotein cholesterol; Crea, creatinine; UA, uric acid; RBC, red blood cell; HGB, hemoglobin; HCT, hematocrit; MCV, mean corpuscular volume; MCH, mean corpuscular hemoglobin; MCHC, mean corpuscular-hemoglobin concentration; RDW-SD, red blood cell distribution width SD; RDW-CV, red blood cell distribution width CV; PLT, platelet; PDW, platelet distribution width; MPV, mean platelet volume; P-LCR, platelet-large cell rate; PCT, plateletcrit; WBC, white blood cell; #NEUT, neutrophil; #LYMPH, lymphocyte; #MONO, monocyte; #BASO, basophil; #EOS, eosinophil; NEUT%, percentage of neutrophils; LYMPH%, percentage of lymphocytes; MONO%, percentage of monocytes; EOS%, percentage of eosinophils; BASO%, percentage of basophils. a Data are mean ± SD, median (interquartile range) for continuous variables, or percentage for categorical variables.

## Abbreviations

BASO%: percentage of basophils
BMI: body mass index
CAD: coronary artery disease
Crea: creatinine
DBP: diastolic blood pressure
EOS%: percentage of eosinophils
FBG: fasting blood glucose
HCT: hematocrit
HDL-C: high density lipoprotein cholesterol
HGB: hemoglobin
LDL-C: low density lipoprotein cholesterol
LYMPH%: percentage of lymphocytes
MACE: major adverse cardiovascular event
MCH: mean corpuscular hemoglobin
MCHC: mean corpuscular-hemoglobin concentration
MCV: mean corpuscular volume
MI: myocardial infarction
MONO%: percentage of monocytes
MPV: mean platelet volume
NEUT%: percentage of neutrophils
PCT: plateletcrit
PDW: platelet distribution width
PLT: platelet
P-LCR: platelet-large cell rate
RBC: red blood cell
RDW-CV: red blood cell distribution width CV
RDW-SD: red blood cell distribution width SD
SBP: systolic blood pressure
TC: total cholesterol
TG: triglyceride
UA: uric acid
WBC: white blood cell
#NEUT: neutrophil
#LYMPH: lymphocyte
#MONO: monocyte
#BASO: basophil
#EOS: eosinophil

## References

[1] Kaya A, Gamsizkan Z, Kaya N, Davran F (2023). The predictive role of laboratory parameters in cardiovascular risk assessment in obese. Medicine.

[2] Gijsberts CM, den Ruijter HM, de Kleijn DPV, Huisman A, Ten Berg MJ, van Wijk RHA (2015). Hematological Parameters Improve Prediction of Mortality and Secondary Adverse Events in Coronary Angiography Patients: A Longitudinal Cohort Study. Medicine.

[3] Abate E, Degef M, Melkie A, Gnanasekeran N, Mehdi M, Tolcha Y (2023). Haematological Parameters in People with Atherosclerotic Cardiovascular Disease versus Those Who are Only at Risk for Cardiovascular Disease: A Comparative Cross-Sectional Study. Diabetes, metabolic syndrome and obesity: targets and therapy.

[4] Lappegard J, Ellingsen TS, Vik A, Skjelbakken T, Brox J, Mathiesen EB (2015). Red cell distribution width and carotid atherosclerosis progression. The Tromso Study. Thrombosis and haemostasis.

[5] Sahin O, Akpek M, Sarli B, Baktir AO, Savas G, Karadavut S (2015). Association of red blood cell distribution width levels with severity of coronary artery disease in patients with non-ST elevation myocardial infarction. Medical principles and practice: international journal of the Kuwait University, Health Science Centre.

[6] Perlstein TS, Weuve J, Pfeffer MA, Beckman JA (2009). Red blood cell distribution width and mortality risk in a community-based prospective cohort. Archives of internal medicine.

[7] Kofink D, Muller SA, Patel RS, Dorresteijn JAN, Berkelmans GFN, de Groot MCH (2018). Routinely measured hematological parameters and prediction of recurrent vascular events in patients with clinically manifest vascular disease. PloS one.

[8] Luo J, Thomassen JQ, Nordestgaard BG, Tybjaerg-Hansen A, Frikke-Schmidt R (2023). Neutrophil counts and cardiovascular disease. European heart journal.

[9] Vinholt PJ, Hvas AM, Frederiksen H, Bathum L, Jorgensen MK, Nybo M (2016). Platelet count is associated with cardiovascular disease, cancer and mortality: A population-based cohort study. Thrombosis research.

[10] Zhang W, Yang R, Yu X, Wang S, Wang X, Mu H (2022). Design, methods and baseline characteristics of the Beijing Hospital Atherosclerosis Study: a prospective dynamic cohort study. Annals of translational medicine.

[11] Yang K, Sun B, Zhang S, Pan Y, Fang J (2023). RDW-SD is Superior to RDW-CV in Reflecting Liver Fibrosis Stage in Patients with Chronic Hepatitis B. Infection and drug resistance.

[12] Jin F, Chang X, Wang X, Xiong H, Wang L, Zhang B (2023). Relationship between red blood cell-related indices and coronary artery calcification. Postgraduate medical journal.

[13] Kara H, Degirmenci S, Bayir A, Ak A, Akinci M, Dogru A (2015). Red cell distribution width and neurological scoring systems in acute stroke patients. Neuropsychiatric disease and treatment.

[14] Sun XP, Chen WM, Sun ZJ, Ding XS, Gao XY, Liang SW (2014). Impact of red blood cell distribution width on long-term mortality in patients with ST-elevation myocardial infarction. Cardiology.

[15] Liu XM, Ma CS, Liu XH, Du X, Kang JP, Zhang Y (2015). Relationship between red blood cell distribution width and intermediate-term mortality in elderly patients after percutaneous coronary intervention. Journal of geriatric cardiology: JGC.

[16] Nunez J, Nunez E, Rizopoulos D, Minana G, Bodi V, Bondanza L (2014). Red blood cell distribution width is longitudinally associated with mortality and anemia in heart failure patients. Circulation journal: official journal of the Japanese Circulation Society.

[17] Su C, Liao LZ, Song Y, Xu ZW, Mei WY (2014). The role of red blood cell distribution width in mortality and cardiovascular risk among patients with coronary artery diseases: a systematic review and meta-analysis. Journal of thoracic disease.

[18] Vieira-Cardoso N, Pereira-Neves A, Fragao-Marques M, Duarte-Gamas L, Domingues-Monteiro D, Vidoedo J (2023). Red blood cell distribution width as a predictor of cardiovascular outcomes in extensive aortoiliac disease. The Journal of cardiovascular surgery.

[19] Patel KV, Mohanty JG, Kanapuru B, Hesdorffer C, Ershler WB, Rifkind JM (2013). Association of the red cell distribution width with red blood cell deformability. Advances in experimental medicine and biology.

[20] Lippi G, Targher G, Montagnana M, Salvagno GL, Zoppini G, Guidi GC (2009). Relation between red blood cell distribution width and inflammatory biomarkers in a large cohort of unselected outpatients. Archives of pathology & laboratory medicine.

[21] Kong P, Cui ZY, Huang XF, Zhang DD, Guo RJ, Han M (2022). Inflammation and atherosclerosis: signaling pathways and therapeutic intervention. Signal transduction and targeted therapy.

[22] Yamaguchi M, Koketsu R, Suzukawa M, Kawakami A, Iikura M (2009). Human basophils and cytokines/chemokines. Allergology international: official journal of the Japanese Society of Allergology.

[23] Schwartz C, Eberle JU, Voehringer D (2016). Basophils in inflammation. European journal of pharmacology.

[24] Wang H, Li H, Wang Y, Zhao C, Tian QW, Wang Q (2023). Hematological parameters and early-onset coronary artery disease: a retrospective case-control study based on 3366 participants. Therapeutic advances in chronic disease.

[25] Bongiovanni D, Han J, Klug M, Kirmes K, Viggiani G, von Scheidt M (2022). Role of Reticulated Platelets in Cardiovascular Disease. Arteriosclerosis, thrombosis, and vascular biology.

[26] Pafili K, Penlioglou T, Mikhailidis DP, Papanas N (2019). Mean platelet volume and coronary artery disease. Current opinion in cardiology.

[27] van Gils JM, Zwaginga JJ, Hordijk PL (2009). Molecular and functional interactions among monocytes, platelets, and endothelial cells and their relevance for cardiovascular diseases. Journal of leukocyte biology.

[28] Ren ZJ, Ren PW, Yang B, Liao J, Liu SZ, Lu DL (2017). Mean platelet volume, platelet distribution width and platelet count in erectile dysfunction: A systematic review and meta-analysis. Andrologia.

[29] Tsai MT, Chen YT, Lin CH, Huang TP, Tarng DC, Taiwan Geriatric Kidney Disease Research G (2015). U-shaped mortality curve associated with platelet count among older people: a community-based cohort study. Blood.

[30] Cetin MS, Ozcan Cetin EH, Akdi A, Aras D, Topaloglu S, Temizhan A (2017). Platelet distribution width and plateletcrit: novel biomarkers of ST elevation myocardial infarction in young patients. Kardiologia polska.

[31] Gawaz M, Vogel S (2013). Platelets in tissue repair: control of apoptosis and interactions with regenerative cells. Blood.

[32] Aslan S, Demir AR, Demir Y, Tasbulak O, Altunova M, Karakayali M (2019). Usefulness of plateletcrit in the prediction of major adverse cardiac and cerebrovascular events in patients with carotid artery stenosis. Vascular.

[33] Gul M, Uyarel H, Akgul O, Akkaya E, Surgit O, Cakmak HA (2016). Long-term prognostic significance of admission plateletcrit values in patients with non-ST elevation myocardial infarction. Blood coagulation & fibrinolysis: an international journal in haemostasis and thrombosis.

[34] Ussher JR, Drucker DJ (2023). Glucagon-like peptide 1 receptor agonists: cardiovascular benefits and mechanisms of action. Nature reviews Cardiology.

[35] Atkins JL, Wannamathee SG (2020). Sarcopenic obesity in ageing: cardiovascular outcomes and mortality. The British journal of nutrition.

[36] Bastien M, Poirier P, Lemieux I, Despres JP (2014). Overview of epidemiology and contribution of obesity to cardiovascular disease. Progress in cardiovascular diseases.

[37] Vidal-Petiot E, Ford I, Greenlaw N, Ferrari R, Fox KM, Tardif JC (2016). Cardiovascular event rates and mortality according to achieved systolic and diastolic blood pressure in patients with stable coronary artery disease: an international cohort study. Lancet.

[38] Beddhu S, Chertow GM, Cheung AK, Cushman WC, Rahman M, Greene T (2018). Influence of Baseline Diastolic Blood Pressure on Effects of Intensive Compared With Standard Blood Pressure Control. Circulation.

[39] Bhatt A, Rohatgi A (2016). HDL Cholesterol Efflux Capacity: Cardiovascular Risk Factor and Potential Therapeutic Target. Current atherosclerosis reports.

[40] Mitchell JD, Laurie M, Xia Q, Dreyfus B, Jain N, Jain A (2023). Risk profiles and incidence of cardiovascular events across different cancer types. ESMO open.

[41] Weir MR (2024). Cardiovascular risk reduction in type 2 diabetes: What the non-specialist needs to know about current guidelines. Diabetes, obesity & metabolism.

[42] Prina E, Ranzani OT, Torres A (2015). Community-acquired pneumonia. Lancet.

[43] Schmittinger CA, Torgersen C, Luckner G, Schroder DC, Lorenz I, Dunser MW (2012). Adverse cardiac events during catecholamine vasopressor therapy: a prospective observational study. Intensive care medicine.

[44] Balogun RA, Omotoso BA, Xin W, Ma JZ, Scully KW, Arogundade FA (2017). Major Depression and Long-Term Outcomes of Acute Kidney Injury. Nephron.

[45] Crozier J, Roig M, Eng JJ, MacKay-Lyons M, Fung J, Ploughman M (2018). High-Intensity Interval Training After Stroke: An Opportunity to Promote Functional Recovery, Cardiovascular Health, and Neuroplasticity. Neurorehabilitation and neural repair.

