# Supplementary material for: Hematological parameters and major adverse cardiovascular events: a prospective study in a Chinese population involving 2,970 participants
Source: Int J Med Sci. 2025 Mar 24;22(8):1924–35. doi: 10.7150/ijms.104118 (PMC11983297; doi:10.7150/ijms.104118)
Supplement: Supplementary file 1 — Supplementary figure and tables. [file ijmsv22p1924s1.pdf]

Supplement Figure 1

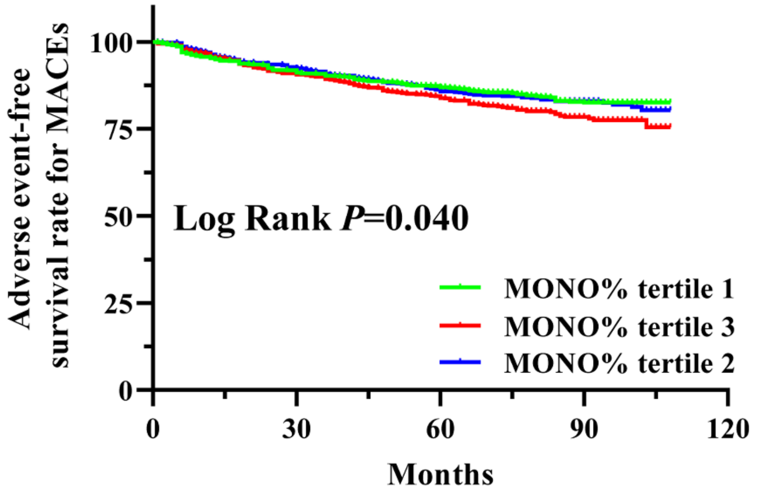

Number at risk

**Tertile 1**	**904**	**824**	**866**	**878**	**904**
**Tertile 2**	**829**	**766**	**775**	**813**	**825**
**Tertile 3**	**845**	**766**	**788**	**813**	**842**

Supplement Figure 1. Kaplan–Meier survival analysis of adverse event-free survival rate for MACEs based on MONO% in this population.

Adverse event-free survival rate for MACEs in this population divided by tertiles of MONO % levels during the follow-up period.

Supplement table 1. The *P* value of Kaplan–Meier survival analysis of MACEs, MI, stroke, revascularization, death, non-cardiovascular death and cardiovascular death based on hematologic indices.

Hematologic indices	MACEs	MI	Stroke	Revascul -arization	Death	Non-cardio-vascular death	cardiovascular death
RBC, 10^{12}/L	0.010	0.424	0.545	0.264	<0.001	<0.001	0.011
HGB, g/L	0.065	0.361	0.380	0.510	<0.001	<0.001	0.024
HCT, %	0.054	0.071	0.702	0.804	<0.001	0.001	0.067
MCV, fL	0.354	0.756	0.170	0.073	0.426	0.296	0.381
MCH, pg	0.481	0.790	0.663	0.261	0.879	0.988	0.815
MCHC, g/L	0.671	0.623	0.336	0.065	0.037	0.086	0.148
RDW-CV, %	0.002	0.523	0.264	0.880	<0.001	<0.001	0.004
RDW-SD, fL	0.012	0.61	0.503	0.051	<0.001	<0.001	0.004
PLT, 10^9/L	<0.001	0.321	0.615	0.557	<0.001	<0.001	0.011
PCT, %	0.002	0.620	0.677	0.192	<0.001	<0.001	0.068
MPV, fL	0.349	0.583	0.600	0.690	0.013	0.390	0.015
PDW, %	0.128	0.420	0.685	0.649	0.070	0.716	0.033
P-LCR, %	0.233	0.563	0.859	0.894	0.014	0.241	0.031
WBC, 10^9/L	0.105	0.405	0.902	0.008	0.122	0.110	0.130
#NEUT, 10^9/L	0.216	0.226	0.493	0.021	0.568	0.808	0.063
#LYMPH, 10^9/L	0.123	0.439	0.851	0.712	<0.001	<0.001	0.033
#MONO, 10^9/L	0.073	0.777	0.900	0.718	0.003	0.114	0.024
#BASO, 10^9/L	0.665	0.625	0.823	0.146	0.094	0.377	0.066
#EOS, 10^9/L	0.880	0.492	0.994	0.462	0.711	0.446	0.983
NEUT%, %	0.024	0.059	0.780	0.158	0.001	0.036	0.015
LYMPH%, %	0.001	0.199	0.294	0.023	<0.001	0.001	0.004
MONO%, %	0.040	0.641	0.658	0.895	0.002	0.057	0.033
BASO%, %	0.018	0.145	0.212	0.041	0.650	0.487	0.937
EOS%, %	0.170	0.732	0.981	0.488	0.064	0.176	0.219

Abbreviations: MACE, major adverse cardiovascular event,; MI, myocardial infarction; RBC, red blood cell; HGB, hemoglobin; HCT, hematocrit; MCV, mean corpuscular volume; MCH, mean corpuscular hemoglobin; MCHC, mean corpuscular-hemoglobin concentration; RDW-SD, red blood cell distribution width SD; RDW-CV, red blood cell distribution width CV; PLT, platelet; PDW, platelet distribution width; MPV, mean platelet volume; P-LCR, platelet-large cell rate; PCT, plateletcrit; WBC, white blood cell; #NEUT, neutrophil; #LYMPH, lymphocyte; #MONO, monocyte; #BASO, eosinophil; #EOS, basophil; NEUT%, percentage of neutrophils; LYMPH%, percentage of lymphocytes; MONO%, percentage of monocytes; EOS%, percentage of eosinophils; BASO%, percentage of basophils.

a Data are mean ± SD, median (interquartile range) for continuous variables, or percentage for categorical variables.

Supplement table 2. Odds ratios (95% confidence intervals) for MACEs according to hematologic indices levels (per 1-SD increment).

Hematologic indices	MACEs				
	Model	T1	T2	T3	P_{trend} [a]
RBC, 10^{12}/L	Model 1 [b]	1.0	0.729 (0.582-0.913)	0.774 (0.621-0.965)	0.019
	Model 2 [c]	1.0	0.664 (0.538-0.819)	1.026 (1.016-1.036)	0.104
	Model 3 [d]	1.0	0.826 (0.650-1.05)	0.86 (0.668-1.108)	0.238
HGB, g/L	Model 1 [b]	1.0	0.779 (0.623-0.975)	0.825 (0.661-1.03)	0.080
	Model 2 [c]	1.0	0.773 (0.611-0.980)	0.817 (0.631-1.057)	0.121
	Model 3 [d]	1.0	0.841 (0.658-1.073)	0.915 (0.701-1.194)	0.514
HCT, %	Model 1 [b]	1.0	0.789 (0.633-0.984)	0.795 (0.634-0.996)	0.041
	Model 2 [c]	1.0	0.796 (0.632-1.003)	0.797 (0.619-1.027)	0.076
	Model 3 [d]	1.0	0.862 (0.679-1.094)	0.864 (0.665-1.124)	0.271
MCV, fL	Model 1 [b]	1.0	0.848 (0.675-1.065)	0.948 (0.761-1.182)	0.625
	Model 2 [c]	1.0	0.807 (0.642-1.014)	0.823 (0.658-1.029)	0.088
	Model 3 [d]	1.0	0.878 (0.694-1.112)	0.907 (0.717-1.148)	0.418
MCH, pg	Model 1 [b]	1.0	0.880 (0.702-1.104)	0.988 (0.790-1.235)	0.904
	Model 2 [c]	1.0	0.844 (0.672-1.059)	0.866 (0.689-1.088)	0.218
	Model 3 [d]	1.0	0.889 (0.702-1.124)	0.952 (0.752-1.206)	0.686
MCHC, g/L	Model 1 [b]	1.0	0.988 (0.789-1.237)	1.087 (0.867-1.362)	0.480
	Model 2 [c]	1.0	0.899 (0.709-1.139)	0.910 (0.724-1.145)	0.380
	Model 3 [d]	1.0	0.857 (0.671-1.093)	0.899 (0.711-1.136)	0.214
RDW-CV, %	Model 1 [b]	1.0	1.117 (0.880-1.417)	1.475 (1.174-1.854)	0.001
	Model 2 [c]	1.0	1.040 (0.818-1.322)	1.290 (1.02-1.631)	0.028
	Model 3 [d]	1.0	1.066 (0.832-1.364)	1.292 (1.013-1.647)	0.034
RDW-SD, fL	Model 1 [b]	1.0	0.874 (0.689-1.108)	1.225 (0.982-1.529)	0.065
	Model 2 [c]	1.0	0.790 (0.621-1.004)	0.985 (0.780-1.242)	0.978
	Model 3 [d]	1.0	0.800 (0.624-1.025)	1.035 (0.813-1.318)	0.712
PLT, 10^9/L	Model 1 [b]	1.0	0.654 (0.521-0.819)	0.720 (0.578-0.897)	0.002
	Model 2 [c]	1.0	0.715 (0.569-0.898)	0.865 (0.689-1.086)	0.153
	Model 3 [d]	1.0	0.721 (0.570-0.912)	0.865 (0.685-1.093)	1.173
PCT, %	Model 1 [b]	1.0	0.676 (0.535-0.853)	0.759 (0.609-0.947)	0.007
	Model 2 [c]	1.0	0.767 (0.605-0.972)	0.915 (0.727-1.150)	0.319
	Model 3 [d]	1.0	0.750 (0.586-0.958)	0.908 (0.719-1.146)	0.296
MPV, fL	Model 1 [b]	1.0	1.063 (0.848-1.334)	1.179 (0.941-1.477)	0.153
	Model 2 [c]	1.0	1.050 (0.837-1.317)	1.161 (0.927-1.455)	0.197
	Model 3 [d]	1.0	1.062 (0.843-1.339)	1.148 (0.910-1.449)	0.245
PDW, %	Model 1 [b]	1.0	1.071 (0.848-1.353)	1.253 (0.999-1.571)	0.050
	Model 2 [c]	1.0	1.077 (0.852-1.36)	1.238 (0.987-1.553)	0.064
	Model 3 [d]	1.0	1.088 (0.856-1.382)	1.215 (0.961-1.535)	0.103

P-LCR, %	Model 1 [b]	1.0	1.146 (0.909-1.445)	1.218 (0.967-1.534)	0.095
	Model 2 [c]	1.0	1.131 (0.897-1.425)	1.189 (0.943-1.498)	0.144
	Model 3 [d]	1.0	1.141 (0.900-1.446)	1.180 (0.929-1.498)	0.175
WBC, 10^9/L	Model 1 [b]	1.0	0.868 (0.689-1.093)	1.108 (0.889-1.383)	0.355
	Model 2 [c]	1.0	0.878 (0.697-1.107)	1.153 (0.922-1.442)	0.211
	Model 3 [d]	1.0	0.890 (0.701-1.13)	1.127 (0.893-1.422)	0.302
#NEUT, 10^9/L	Model 1 [b]	1.0	1.096 (0.871-1.381)	1.223 (0.974-1.534)	0.082
	Model 2 [c]	1.0	1.068 (0.847-1.347)	1.193 (0.949-1.501)	0.129
	Model 3 [d]	1.0	1.054 (0.830-1.340)	1.133 (0.893-1.437)	0.303
#LYMPH, 10^9/L	Model 1 [b]	1.0	0.831 (0.666-1.037)	0.810 (0.647-1.014)	0.063
	Model 2 [c]	1.0	0.932 (0.744-1.166)	0.987 (0.781-1.247)	0.888
	Model 3 [d]	1.0	0.882 (0.699-1.111)	0.975 (0.768-1.239)	0.796
#MONO, 10^9/L	Model 1 [b]	1.0	0.931 (0.739-1.173)	1.198 (0.960-1.496)	0.111
	Model 2 [c]	1.0	0.899 (0.713-1.133)	1.094 (0.872-1.374)	0.431
	Model 3 [d]	1.0	0.901 (0.710-1.143)	1.073 (0.848-1.357)	0.552
#BASO, 10^9/L	Model 1 [b]	1.0	0.947 (0.735-1.220)	0.904 (0.722-1.133)	0.368
	Model 2 [c]	1.0	0.934 (0.724-1.203)	0.911 (0.727-1.142)	0.393
	Model 3 [d]	1.0	0.898 (0.691-1.168)	0.921 (0.732-1.160)	0.422
#EOS, 10^9/L	Model 1 [b]	1.0	1.036 (0.826-1.300)	1.058 (0.848-1.320)	0.616
	Model 2 [c]	1.0	1.030 (0.821-1.292)	1.034 (0.827-1.291)	0.769
	Model 3 [d]	1.0	1.049 (0.831-1.325)	1.048 (0.832-1.318)	0.687
NEUT%, %	Model 1 [b]	1.0	1.055 (0.835-1.335)	1.332 (1.065-1.666)	0.011
	Model 2 [c]	1.0	0.993 (0.785-1.257)	1.177 (0.937-1.477)	0.149
	Model 3 [d]	1.0	0.945 (0.741-1.205)	1.145 (0.905-1.447)	0.235
LYMPH%, %	Model 1 [b]	1.0	0.790 (0.636-0.982)	0.644 (0.512-0.810)	<0.001
	Model 2 [c]	1.0	0.866 (0.696-1.079)	0.762 (0.602-0.965)	0.023
	Model 3 [d]	1.0	0.854 (0.681-1.071)	0.780 (0.612-0.995)	0.042
MONO%, %	Model 1 [b]	1.0	1.049 (0.830-1.326)	1.304 (1.044-1.628)	0.019
	Model 2 [c]	1.0	0.986 (0.779-1.247)	1.128 (0.899-1.416)	0.286
	Model 3 [d]	1.0	1.010 (0.793-1.285)	1.159 (0.917-1.466)	0.210
BASO%, %	Model 1 [b]	1.0	0.801 (0.636-1.009)	0.737 (0.584-0.931)	0.006
	Model 2 [c]	1.0	0.809 (0.642-1.019)	0.731 (0.578-0.923)	0.005
	Model 3 [d]	1.0	0.772 (0.607-0.98)	0.750 (0.591-0.953)	0.009
EOS%, %	Model 1 [b]	1.0	0.815 (0.647-1.028)	0.989 (0.797-1.227)	0.873
	Model 2 [c]	1.0	0.805 (0.638-1.015)	0.962 (0.775-1.194)	0.686
	Model 3 [d]	1.0	0.834 (0.657-1.058)	0.998 (0.799-1.248)	0.957

[a] *P* values for trend.

[b] Model 1: Crude risk.

[c] Model 2: Adjusted for age and gender.

[d] Model 3: Further adjusted for smoking status, obesity or overweight, hypertension, dyslipidemia, diabetes, stroke and family history of premature CAD.

Supplement table 3. Odds ratios (95% confidence intervals) for MI according to hematologic indices levels (per 1-SD increment).

Hematologic indices	MI				
	Model	T1	T2	T3	P_{trend} [a]
RBC, 10^{12}/L	Model 1 [b]	1.0	1.176 (0.605-2.286)	0.728 (0.345-1.540)	0.427
	Model 2 [c]	1.0	1.100 (0.551-2.192)	0.669 (0.291-1.541)	0.361
	Model 3 [d]	1.0	1.117 (0.549-2.275)	0.728 (0.312-1.699)	0.476
HGB, g/L	Model 1 [b]	1.0	0.991 (0.516-1.905)	0.612 (0.289-1.296)	0.214
	Model 2 [c]	1.0	0.943 (0.473-1.880)	0.554 (0.232-1.324)	0.199
	Model 3 [d]	1.0	1.082 (0.530-2.206)	0.666 (0.274-1.620)	0.395
HCT, %	Model 1 [b]	1.0	1.453 (0.758-2.785)	0.610 (0.267-1.394)	0.301
	Model 2 [c]	1.0	1.401 (0.709-2.766)	0.576 (0.230-1.439)	0.297
	Model 3 [d]	1.0	1.643 (0.804-3.359)	0.710 (0.276-1.826)	0.552
MCV, fL	Model 1 [b]	1.0	0.765 (0.375-1.561)	0.928 (0.473-1.821)	0.816
	Model 2 [c]	1.0	0.793 (0.388-1.621)	1.041 (0.525-2.067)	0.935
	Model 3 [d]	1.0	0.898 (0.429-1.879)	1.236 (0.602-2.536)	0.576
MCH, pg	Model 1 [b]	1.0	0.869 (0.424-1.781)	1.113 (0.562-2.203)	0.757
	Model 2 [c]	1.0	0.926 (0.450-1.904)	1.295 (0.641-2.616)	0.479
	Model 3 [d]	1.0	1.087 (0.515-2.294)	1.522 (0.730-3.175)	0.263
MCHC, g/L	Model 1 [b]	1.0	0.724 (0.355-1.479)	0.986 (0.503-1.933)	0.942
	Model 2 [c]	1.0	0.741 (0.359-1.530)	1.036 (0.508-2.111)	0.946
	Model 3 [d]	1.0	0.809 (0.384-1.703)	1.107 (0.533-2.303)	0.789
RDW-CV, %	Model 1 [b]	1.0	1.458 (0.725-2.931)	1.104 (0.519-2.350)	0.804
	Model 2 [c]	1.0	1.541 (0.763-3.112)	1.224 (0.567-2.642)	0.602
	Model 3 [d]	1.0	1.362 (0.664-2.795)	1.236 (0.572-2.672)	0.585
RDW-SD, fL	Model 1 [b]	1.0	1.053 (0.543-2.044)	0.740 (0.354-1.550)	0.445
	Model 2 [c]	1.0	1.123 (0.575-2.192)	0.855 (0.398-1.836)	0.725
	Model 3 [d]	1.0	1.133 (0.566-2.267)	0.951 (0.435-2.080)	0.929
PLT, 10^{9}/L	Model 1 [b]	1.0	1.116 (0.580-2.148)	0.642 (0.301-1.371)	0.276
	Model 2 [c]	1.0	1.003 (0.518-1.941)	0.506 (0.231-1.109)	0.094
	Model 3 [d]	1.0	1.071 (0.546-2.101)	0.496 (0.219-1.125)	0.100
PCT, %	Model 1 [b]	1.0	0.825 (0.416-1.638)	0.711 (0.352-1.438)	0.333
	Model 2 [c]	1.0	0.708 (0.352-1.423)	0.554 (0.265-1.158)	0.110
	Model 3 [d]	1.0	0.776 (0.383-1.572)	0.544 (0.254-1.166)	0.116
MPV, fL	Model 1 [b]	1.0	1.266 (0.618-2.594)	1.449 (0.714-2.940)	0.303
	Model 2 [c]	1.0	1.285 (0.627-2.634)	1.465 (0.720-2.977)	0.291
	Model 3 [d]	1.0	1.202 (0.578-2.497)	1.401 (0.682-2.880)	0.358
PDW, %	Model 1 [b]	1.0	1.576 (0.759-3.273)	1.513 (0.722-3.168)	0.284
	Model 2 [c]	1.0	1.576 (0.759-3.272)	1.525 (0.727-3.196)	0.274
	Model 3 [d]	1.0	1.486 (0.709-3.115)	1.462 (0.690-3.096)	0.329

P-LCR, %	Model 1 [b]	1.0	1.256 (0.604-2.611)	1.475 (0.722-3.009)	0.286
	Model 2 [c]	1.0	1.270 (0.610-2.640)	1.494 (0.730-3.056)	0.272
	Model 3 [d]	1.0	1.196 (0.568-2.520)	1.448 (0.701-2.991)	0.316
WBC, 10^9/L	Model 1 [b]	1.0	1.618 (0.791-3.310)	1.265 (0.592-2.703)	0.567
	Model 2 [c]	1.0	1.647 (0.802-3.381)	1.279 (0.591-2.766)	0.557
	Model 3 [d]	1.0	1.718 (0.810-3.642)	1.441 (0.648-3.206)	0.394
#NEUT, 10^9/L	Model 1 [b]	1.0	0.672 (0.312-1.448)	1.273 (0.660-2.456)	0.443
	Model 2 [c]	1.0	0.707 (0.327-1.530)	1.342 (0.687-2.622)	0.365
	Model 3 [d]	1.0	0.662 (0.297-1.475)	1.259 (0.633-2.503)	0.481
#LYMPH, 10^9/L	Model 1 [b]	1.0	0.672 (0.321-1.407)	1.050 (0.541-2.038)	0.875
	Model 2 [c]	1.0	0.609 (0.288-1.286)	0.904 (0.456-1.789)	0.813
	Model 3 [d]	1.0	0.604 (0.277-1.314)	1.015 (0.504-2.043)	0.917
#MONO, 10^9/L	Model 1 [b]	1.0	0.778 (0.387-1.564)	0.916 (0.462-1.819)	0.782
	Model 2 [c]	1.0	0.822 (0.406-1.662)	1.032 (0.506-2.105)	0.962
	Model 3 [d]	1.0	0.784 (0.378-1.628)	1.073 (0.519-2.218)	0.879
#BASO, 10^9/L	Model 1 [b]	1.0	0.666 (0.277-1.602)	0.834 (0.418-1.667)	0.510
	Model 2 [c]	1.0	0.678 (0.282-1.630)	0.842 (0.420-1.684)	0.531
	Model 3 [d]	1.0	0.728 (0.301-1.763)	0.925 (0.458-1.868)	0.735
#EOS, 10^9/L	Model 1 [b]	1.0	0.922 (0.436-1.949)	1.365 (0.702-2.655)	0.357
	Model 2 [c]	1.0	0.943 (0.446-1.996)	1.431 (0.731-2.799)	0.296
	Model 3 [d]	1.0	0.952 (0.436-2.079)	1.607 (0.807-3.199)	0.175
NEUT%, %	Model 1 [b]	1.0	0.431 (0.189-0.985)	1.093 (0.584-2.049)	0.776
	Model 2 [c]	1.0	0.452 (0.198-1.035)	1.205 (0.639-2.272)	0.580
	Model 3 [d]	1.0	0.385 (0.161-0.918)	1.098 (0.573-2.104)	0.802
LYMPH%, %	Model 1 [b]	1.0	0.543 (0.251-1.177)	1.037 (0.544-1.975)	0.898
	Model 2 [c]	1.0	0.504 (0.232-1.095)	0.891 (0.460-1.725)	0.771
	Model 3 [d]	1.0	0.413 (0.179-0.956)	0.929 (0.476-1.814)	0.870
MONO%, %	Model 1 [b]	1.0	1.238 (0.631-2.427)	0.889 (0.427-1.849)	0.781
	Model 2 [c]	1.0	1.322 (0.671-2.605)	1.029 (0.487-2.176)	0.895
	Model 3 [d]	1.0	1.346 (0.674-2.688)	0.978 (0.449-2.133)	0.995
BASO%, %	Model 1 [b]	1.0	0.538 (0.246-1.174)	0.572 (0.271-1.206)	0.087
	Model 2 [c]	1.0	0.537 (0.246-1.172)	0.580 (0.275-1.224)	0.093
	Model 3 [d]	1.0	0.578 (0.263-1.272)	0.639 (0.301-1.358)	0.167
EOS%, %	Model 1 [b]	1.0	0.970 (0.466-2.016)	1.248 (0.636-2.447)	0.518
	Model 2 [c]	1.0	0.983 (0.473-2.044)	1.296 (0.659-2.547)	0.453
	Model 3 [d]	1.0	1.003 (0.468-2.148)	1.439 (0.720-2.872)	0.301

[a] *P* values for trend.

[b] Model 1: Crude risk.

[c] Model 2: Adjusted for age and gender.

[d] Model 3: Further adjusted for smoking status, obesity or overweight, hypertension, dyslipidemia, diabetes, stroke and family history of premature CAD.

Supplement table 4. Odds ratios (95% confidence intervals) for stroke according to hematologic indices levels (per 1-SD increment).

Hematologic indices	Stroke				
	Model	T1	T2	T3	P_{trend}[a]
RBC, 10^{12}/L	Model 1 [b]	1.0	0.988 (0.580-1.685)	0.750 (0.424-1.326)	0.329
	Model 2 [c]	1.0	1.143 (0.657-1.990)	0.933 (0.500-1.742)	0.848
	Model 3 [d]	1.0	1.230 (0.696-2.172)	0.904 (0.477-1.713)	0.764
HGB, g/L	Model 1 [b]	1.0	1.194 (0.702-2.030)	0.801 (0.445-1.442)	0.482
	Model 2 [c]	1.0	1.331 (0.761-2.328)	0.977 (0.501-1.905)	0.980
	Model 3 [d]	1.0	1.407 (0.791-2.504)	1.044 (0.527-2.065)	0.890
HCT, %	Model 1 [b]	1.0	0.939 (0.551-1.600)	0.787 (0.445-1.392)	0.415
	Model 2 [c]	1.0	1.041 (0.597-1.815)	0.934 (0.496-1.759)	0.842
	Model 3 [d]	1.0	1.120 (0.632-1.985)	0.955 (0.500-1.824)	0.896
MCV, fL	Model 1 [b]	1.0	0.578 (0.320-1.045)	0.911 (0.544-1.526)	0.686
	Model 2 [c]	1.0	0.550 (0.304-0.995)	0.770 (0.455-1.302)	0.320
	Model 3 [d]	1.0	0.655 (0.358-1.199)	0.949 (0.549-1.638)	0.836
MCH, pg	Model 1 [b]	1.0	0.778 (0.445-1.359)	0.940 (0.548-1.612)	0.806
	Model 2 [c]	1.0	0.751 (0.429-1.316)	0.840 (0.483-1.460)	0.529
	Model 3 [d]	1.0	0.872 (0.492-1.546)	1.031 (0.584-1.822)	0.924
MCHC, g/L	Model 1 [b]	1.0	1.472 (0.833-2.603)	1.461 (0.816-2.618)	0.207
	Model 2 [c]	1.0	1.625 (0.911-2.899)	1.706 (0.926-3.145)	0.088
	Model 3 [d]	1.0	1.632 (0.902-2.952)	1.797 (0.967-3.341)	0.065
RDW-CV, %	Model 1 [b]	1.0	1.563 (0.871-2.805)	1.533 (0.844-2.785)	0.174
	Model 2 [c]	1.0	1.404 (0.779-2.531)	1.258 (0.682-2.320)	0.511
	Model 3 [d]	1.0	1.411 (0.773-2.576)	1.253 (0.669-2.346)	0.536
RDW-SD, fL	Model 1 [b]	1.0	0.947 (0.528-1.698)	1.285 (0.740-2.231)	0.364
	Model 2 [c]	1.0	0.837 (0.464-1.511)	0.989 (0.554-1.763)	0.998
	Model 3 [d]	1.0	0.912 (0.497-1.673)	1.187 (0.656-2.147)	0.551
PLT, 10^9/L	Model 1 [b]	1.0	0.776 (0.439-1.372)	0.998 (0.585-1.701)	0.994
	Model 2 [c]	1.0	0.862 (0.485-1.533)	1.238 (0.713-2.150)	0.464
	Model 3 [d]	1.0	0.850 (0.471-1.533)	1.220 (0.698-2.134)	0.492
PCT, %	Model 1 [b]	1.0	0.845 (0.485-1.472)	0.791 (0.454-1.378)	0.393
	Model 2 [c]	1.0	0.962 (0.547-1.691)	0.957 (0.538-1.702)	0.876
	Model 3 [d]	1.0	1.024 (0.576-1.821)	0.983 (0.547-1.770)	0.964
MPV, fL	Model 1 [b]	1.0	1.026 (0.607-1.732)	0.776 (0.435-1.383)	0.415
	Model 2 [c]	1.0	1.001 (0.592-1.691)	0.742 (0.415-1.325)	0.333
	Model 3 [d]	1.0	1.054 (0.620-1.791)	0.745 (0.411-1.349)	0.361
PDW, %	Model 1 [b]	1.0	0.779 (0.442-1.371)	0.905 (0.524-1.561)	0.701
	Model 2 [c]	1.0	0.783 (0.445-1.379)	0.878 (0.508-1.517)	0.627
	Model 3 [d]	1.0	0.786 (0.443-1.393)	0.857 (0.490-1.499)	0.578

P-LCR, %	Model 1 [b]	1.0	0.898 (0.518-1.556)	0.859 (0.489-1.509)	0.594
	Model 2 [c]	1.0	0.883 (0.509-1.530)	0.819 (0.466-1.441)	0.486
	Model 3 [d]	1.0	0.895 (0.513-1.560)	0.805 (0.452-1.435)	0.461
WBC, 10^9/L	Model 1 [b]	1.0	0.883 (0.507-1.538)	0.968 (0.559-1.676)	0.904
	Model 2 [c]	1.0	0.915 (0.525-1.597)	1.047 (0.601-1.821)	0.878
	Model 3 [d]	1.0	0.835 (0.475-1.467)	0.869 (0.495-1.526)	0.624
#NEUT, 10^9/L	Model 1 [b]	1.0	1.095 (0.645-1.860)	0.780 (0.435-1.397)	0.422
	Model 2 [c]	1.0	1.093 (0.642-1.861)	0.787 (0.437-1.417)	0.442
	Model 3 [d]	1.0	0.929 (0.539-1.600)	0.661 (0.365-1.197)	0.173
#LYMPH, 10^9/L	Model 1 [b]	1.0	0.949 (0.539-1.670)	1.108 (0.640-1.921)	0.707
	Model 2 [c]	1.0	1.111 (0.627-1.969)	1.436 (0.813-2.536)	0.212
	Model 3 [d]	1.0	1.022 (0.574-1.820)	1.328 (0.750-2.350)	0.334
#MONO, 10^9/L	Model 1 [b]	1.0	0.922 (0.529-1.605)	1.050 (0.606-1.818)	0.869
	Model 2 [c]	1.0	0.904 (0.518-1.578)	0.986 (0.562-1.730)	0.956
	Model 3 [d]	1.0	0.835 (0.475-1.468)	0.929 (0.525-1.643)	0.790
#BASO, 10^9/L	Model 1 [b]	1.0	0.843 (0.435-1.633)	1.062 (0.625-1.804)	0.905
	Model 2 [c]	1.0	0.841 (0.434-1.630)	1.090 (0.642-1.853)	0.835
	Model 3 [d]	1.0	0.794 (0.408-1.546)	1.051 (0.611-1.808)	0.967
#EOS, 10^9/L	Model 1 [b]	1.0	1.007 (0.580-1.749)	0.978 (0.567-1.687)	0.939
	Model 2 [c]	1.0	1.019 (0.587-1.772)	0.980 (0.566-1.696)	0.946
	Model 3 [d]	1.0	1.040 (0.591-1.828)	1.039 (0.596-1.812)	0.890
NEUT%, %	Model 1 [b]	1.0	0.912 (0.532-1.565)	0.818 (0.468-1.430)	0.481
	Model 2 [c]	1.0	0.862 (0.502-1.481)	0.710 (0.403-1.250)	0.235
	Model 3 [d]	1.0	0.762 (0.438-1.327)	0.660 (0.372-1.173)	0.156
LYMPH%, %	Model 1 [b]	1.0	0.653 (0.365-1.169)	0.971 (0.575-1.640)	0.918
	Model 2 [c]	1.0	0.738 (0.410-1.327)	1.189 (0.691-2.045)	0.552
	Model 3 [d]	1.0	0.795 (0.440-1.436)	1.330 (0.764-2.314)	0.341
MONO%, %	Model 1 [b]	1.0	0.971 (0.547-1.722)	1.225 (0.714-2.101)	0.460
	Model 2 [c]	1.0	0.917 (0.515-1.632)	1.057 (0.608-1.838)	0.836
	Model 3 [d]	1.0	1.022 (0.569-1.835)	1.181 (0.670-2.083)	0.562
BASO%, %	Model 1 [b]	1.0	0.567 (0.299-1.076)	0.913 (0.537-1.554)	0.524
	Model 2 [c]	1.0	0.575 (0.303-1.090)	0.909 (0.534-1.548)	0.522
	Model 3 [d]	1.0	0.535 (0.275-1.043)	0.939 (0.550-1.602)	0.580
EOS%, %	Model 1 [b]	1.0	0.976 (0.562-1.695)	0.947 (0.549-1.633)	0.845
	Model 2 [c]	1.0	0.977 (0.563-1.697)	0.933 (0.540-1.611)	0.804
	Model 3 [d]	1.0	0.990 (0.563-1.742)	1.035 (0.595-1.800)	0.906

[a] *P* values for trend.

[b] Model 1: Crude risk.

[c] Model 2: Adjusted for age and gender.

[d] Model 3: Further adjusted for smoking status, obesity or overweight, hypertension, dyslipidemia, diabetes, stroke and family history of premature CAD.

Supplement table 5. Odds ratios (95% confidence intervals) for revascularization according to hematologic indices levels (per 1-SD increment).

Hematologic indices	Revascularization				
	Model	T1	T2	T3	P_{trend} [a]
RBC, 10^{12}/L	Model 1 [b]	1.0	1.107 (0.782-1.569)	1.311 (0.938-1.833)	0.109
	Model 2 [c]	1.0	0.959 (0.669-1.375)	0.999 (0.688-1.451)	0.982
	Model 3 [d]	1.0	0.954 (0.661-1.377)	0.991 (0.678-1.449)	0.984
HGB, g/L	Model 1 [b]	1.0	1.111 (0.790-1.564)	1.220 (0.871-1.708)	0.247
	Model 2 [c]	1.0	0.895 (0.624-1.283)	0.831 (0.563-1.225)	0.354
	Model 3 [d]	1.0	0.900 (0.622-1.303)	0.886 (0.596-1.318)	0.569
HCT, %	Model 1 [b]	1.0	1.026 (0.733-1.437)	1.114 (0.795-1.559)	0.529
	Model 2 [c]	1.0	0.838 (0.590-1.192)	0.777 (0.532-1.135)	0.201
	Model 3 [d]	1.0	0.858 (0.599-1.229)	0.808 (0.548-1.191)	0.291
MCV, fL	Model 1 [b]	1.0	0.800 (0.579-1.105)	0.683 (0.489-0.956)	0.024
	Model 2 [c]	1.0	0.795 (0.575-1.099)	0.679 (0.483-0.955)	0.024
	Model 3 [d]	1.0	0.858 (0.616-1.195)	0.754 (0.530-1.074)	0.115
MCH, pg	Model 1 [b]	1.0	0.819 (0.592-1.133)	0.771 (0.552-1.078)	0.122
	Model 2 [c]	1.0	0.774 (0.559-1.072)	0.686 (0.488-0.966)	0.029
	Model 3 [d]	1.0	0.820 (0.587-1.145)	0.753 (0.531-1.068)	0.108
MCHC, g/L	Model 1 [b]	1.0	1.083 (0.766-1.530)	1.444 (1.036-2.014)	0.029
	Model 2 [c]	1.0	0.974 (0.685-1.385)	1.207 (0.853-1.708)	0.268
	Model 3 [d]	1.0	1.035 (0.721-1.486)	1.275 (0.893-1.822)	0.166
RDW-CV, %	Model 1 [b]	1.0	1.020 (0.729-1.426)	1.086 (0.776-1.521)	0.63
	Model 2 [c]	1.0	1.058 (0.755-1.483)	1.176 (0.833-1.660)	0.357
	Model 3 [d]	1.0	1.026 (0.729-1.445)	1.124 (0.792-1.595)	0.515
RDW-SD, fL	Model 1 [b]	1.0	0.829 (0.602-1.142)	0.651 (0.460-0.921)	0.015
	Model 2 [c]	1.0	0.833 (0.603-1.151)	0.650 (0.452-0.933)	0.019
	Model 3 [d]	1.0	0.800 (0.573-1.115)	0.688 (0.476-0.993)	0.042
PLT, 10^9/L	Model 1 [b]	1.0	0.834 (0.595-1.169)	0.953 (0.688-1.322)	0.767
	Model 2 [c]	1.0	0.857 (0.610-1.204)	1.010 (0.719-1.418)	0.971
	Model 3 [d]	1.0	0.855 (0.605-1.209)	0.991 (0.702-1.399)	0.948
PCT, %	Model 1 [b]	1.0	0.728 (0.512-1.036)	0.954 (0.693-1.312)	0.664
	Model 2 [c]	1.0	0.753 (0.527-1.077)	1.028 (0.737-1.434)	0.978
	Model 3 [d]	1.0	0.731 (0.506-1.055)	1.017 (0.727-1.423)	0.967
MPV, fL	Model 1 [b]	1.0	0.904 (0.648-1.263)	1.048 (0.755-1.456)	0.805
	Model 2 [c]	1.0	0.915 (0.655-1.278)	1.096 (0.789-1.523)	0.616
	Model 3 [d]	1.0	0.922 (0.656-1.294)	1.106 (0.791-1.547)	0.583
PDW, %	Model 1 [b]	1.0	1.115 (0.793-1.569)	1.171 (0.834-1.643)	0.362
	Model 2 [c]	1.0	1.119 (0.795-1.574)	1.209 (0.861-1.697)	0.274
	Model 3 [d]	1.0	1.098 (0.776-1.555)	1.196 (0.847-1.689)	0.309
P-LCR, %	Model 1 [b]	1.0	0.983 (0.700-1.379)	1.062 (0.759-1.486)	0.728

	Model 2 [c]	1.0	0.991 (0.706-1.391)	1.109 (0.792-1.554)	0.55
	Model 3 [d]	1.0	1.002 (0.710-1.414)	1.124 (0.797-1.586)	0.507
WBC, 10^9/L	Model 1 [b]	1.0	0.907 (0.634-1.297)	1.460 (1.054-2.023)	0.018
	Model 2 [c]	1.0	0.856 (0.598-1.227)	1.320 (0.947-1.839)	0.078
	Model 3 [d]	1.0	0.885 (0.612-1.279)	1.349 (0.959-1.898)	0.062
#NEUT, 10^9/L	Model 1 [b]	1.0	1.137 (0.797-1.623)	1.561 (1.116-2.183)	0.008
	Model 2 [c]	1.0	1.064 (0.744-1.522)	1.412 (1.005-1.984)	0.04
	Model 3 [d]	1.0	1.078 (0.746-1.556)	1.408 (0.993-1.997)	0.046
#LYMPH, 10^9/L	Model 1 [b]	1.0	1.151 (0.823-1.608)	1.088(0.773-1.531)	0.635
	Model 2 [c]	1.0	1.130 (0.805-1.586)	1.059 (0.743-1.509)	0.765
	Model 3 [d]	1.0	1.105 (0.781-1.563)	1.103 (0.770-1.579)	0.6
#MONO, 10^9/L	Model 1 [b]	1.0	1.119 (0.802-1.561)	1.138 (0.811-1.597)	0.453
	Model 2 [c]	1.0	1.046 (0.748-1.462)	0.985 (0.696-1.394)	0.928
	Model 3 [d]	1.0	1.046 (0.743-1.473)	1.000 (0.702-1.423)	0.994
#BASO, 10^9/L	Model 1 [b]	1.0	0.677 (0.447-1.025)	0.843 (0.607-1.171)	0.189
	Model 2 [c]	1.0	0.655 (0.433-0.992)	0.804 (0.578-1.118)	0.11
	Model 3 [d]	1.0	0.639 (0.418-0.975)	0.826 (0.592-1.151)	0.148
#EOS, 10^9/L	Model 1 [b]	1.0	1.059 (0.752-1.491)	1.221 (0.882-1.689)	0.23
	Model 2 [c]	1.0	1.020 (0.724-1.437)	1.136 (0.820-1.575)	0.444
	Model 3 [d]	1.0	1.061 (0.749-1.503)	1.152 (0.825-1.609)	0.407
NEUT%, %	Model 1 [b]	1.0	1.057 (0.747-1.497)	1.347 (0.968-1.873)	0.074
	Model 2 [c]	1.0	1.037 (0.731-1.469)	1.324 (0.948-1.850)	0.095
	Model 3 [d]	1.0	0.988 (0.691-1.412)	1.296 (0.921-1.823)	0.127
LYMPH%, %	Model 1 [b]	1.0	0.944 (0.689-1.293)	0.631 (0.445-0.896)	0.011
	Model 2 [c]	1.0	0.935 (0.681-1.285)	0.649 (0.453-0.929)	0.02
	Model 3 [d]	1.0	0.918 (0.664-1.270)	0.668 (0.464-0.962)	0.033
MONO%, %	Model 1 [b]	1.0	0.963 (0.689-1.348)	1.045 (0.752-1.450)	0.8
	Model 2 [c]	1.0	0.918 (0.655-1.286)	0.975 (0.697-1.364)	0.879
	Model 3 [d]	1.0	0.890 (0.632-1.253)	0.964 (0.685-1.355)	0.823
BASO%, %	Model 1 [b]	1.0	0.793 (0.566-1.112)	0.647 (0.454-0.923)	0.012
	Model 2 [c]	1.0	0.780 (0.556-1.093)	0.640 (0.448-0.913)	0.01
	Model 3 [d]	1.0	0.721 (0.509-1.022)	0.636 (0.444-0.912)	0.008
EOS%, %	Model 1 [b]	1.0	0.863 (0.612-1.218)	1.064 (0.773-1.463)	0.721
	Model 2 [c]	1.0	0.844 (0.598-1.191)	1.022 (0.742-1.407)	0.908
	Model 3 [d]	1.0	0.867 (0.611-1.230)	1.039 (0.750-1.441)	0.826

[a] *P* values for trend.

[b] Model 1: Crude risk.

[c] Model 2: Adjusted for age and gender.

[d] Model 3: Further adjusted for smoking status, obesity or overweight, hypertension, dyslipidemia, diabetes, stroke and family history of premature CAD.

Supplement table 6. Odds ratios (95% confidence intervals) for all-cause deaths according to hematologic indices levels (per 1-SD increment).

Hematologic indices	Death				
	Model	T1	T2	T3	P_{trend}[a]
RBC, 10^{12}/L	Model 1 [b]	1.0	0.391 (0.274-0.560)	0.500 (0.360-0.692)	<0.001
	Model 2 [c]	1.0	0.508 (0.352-0.735)	0.746 (0.523-1.064)	0.048
	Model 3 [d]	1.0	0.597 (0.410-0.870)	0.811 (0.559-1.176)	0.166
HGB, g/L	Model 1 [b]	1.0	0.483 (0.342-0.682)	0.567 (0.408-0.787)	<0.001
	Model 2 [c]	1.0	0.580 (0.405-0.832)	0.795 (0.548-1.153)	0.143
	Model 3 [d]	1.0	0.646 (0.444-0.942)	0.881 (0.598-1.298)	0.396
HCT, %	Model 1 [b]	1.0	0.530 (0.379-0.740)	0.559 (0.400-0.783)	<0.001
	Model 2 [c]	1.0	0.652 (0.462-0.921)	0.780 (0.541-1.127)	0.123
	Model 3 [d]	1.0	0.714 (0.497-1.026)	0.855 (0.581-1.256)	0.329
MCV, fL	Model 1 [b]	1.0	1.126 (0.796-1.592)	1.252 (0.893-1.754)	0.192
	Model 2 [c]	1.0	1.023 (0.723-1.448)	0.901 (0.640-1.270)	0.534
	Model 3 [d]	1.0	1.048 (0.730-1.505)	0.913 (0.635-1.312)	0.596
MCH, pg	Model 1 [b]	1.0	0.966 (0.689-1.356)	1.054 (0.753-1.475)	0.762
	Model 2 [c]	1.0	0.914 (0.650-1.285)	0.852 (0.604-1.202)	0.362
	Model 3 [d]	1.0	0.889 (0.623-1.269)	0.861 (0.602-1.231)	0.413
MCHC, g/L	Model 1 [b]	1.0	0.729 (0.527-1.008)	0.666 (0.472-0.940)	0.016
	Model 2 [c]	1.0	0.838 (0.602-1.165)	0.813 (0.568-1.165)	0.235
	Model 3 [d]	1.0	0.832 (0.589-1.173)	0.794 (0.547-1.154)	0.206
RDW-CV, %	Model 1 [b]	1.0	1.317 (0.886-1.956)	2.471 (1.724-3.542)	<0.001
	Model 2 [c]	1.0	1.053 (0.707-1.569)	1.659 (1.147-2.400)	0.003
	Model 3 [d]	1.0	1.182 (0.774-1.806)	1.759 (1.183-2.614)	0.002
RDW-SD, fL	Model 1 [b]	1.0	0.965 (0.643-1.449)	2.419 (1.717-3.409)	<0.001
	Model 2 [c]	1.0	0.748 (0.496-1.127)	1.438 (1.004-2.060)	0.011
	Model 3 [d]	1.0	0.775 (0.505-1.190)	1.428 (0.975-2.091)	0.022
PLT, 10^9/L	Model 1 [b]	1.0	0.478 (0.341-0.670)	0.470 (0.335-0.661)	<0.001
	Model 2 [c]	1.0	0.563 (0.400-0.792)	0.664 (0.468-0.942)	0.008
	Model 3 [d]	1.0	0.565 (0.395-0.807)	0.658 (0.459-0.944)	0.009
PCT, %	Model 1 [b]	1.0	0.487 (0.339-0.698)	0.552 (0.394-0.774)	<0.001
	Model 2 [c]	1.0	0.621 (0.431-0.895)	0.774 (0.547-1.095)	0.071
	Model 3 [d]	1.0	0.564 (0.381-0.833)	0.739 (0.517-1.056)	0.039
MPV, fL	Model 1 [b]	1.0	1.343 (0.943-1.913)	1.665 (1.181-2.347)	0.004
	Model 2 [c]	1.0	1.287 (0.904-1.834)	1.558 (1.104-2.198)	0.011
	Model 3 [d]	1.0	1.295 (0.899-1.866)	1.550 (1.084-2.214)	0.016
PDW, %	Model 1 [b]	1.0	1.228 (0.860-1.753)	1.491 (1.059-2.100)	0.022
	Model 2 [c]	1.0	1.253 (0.878-1.790)	1.425 (1.011-2.009)	0.044
	Model 3 [d]	1.0	1.290 (0.893-1.862)	1.415 (0.988-2.027)	0.059

P-LCR, %	Model 1 [b]	1.0	1.532 (1.068-2.198)	1.667 (1.165-2.385)	0.006
	Model 2 [c]	1.0	1.489 (1.038-2.137)	1.536 (1.072-2.199)	0.023
	Model 3 [d]	1.0	1.483 (1.022-2.151)	1.528 (1.052-2.217)	0.029
WBC, 10^9/L	Model 1 [b]	1.0	0.701 (0.498-0.986)	0.867 (0.625-1.202)	0.367
	Model 2 [c]	1.0	0.752 (0.534-1.059)	1.016 (0.732-1.410)	0.994
	Model 3 [d]	1.0	0.818 (0.572-1.168)	1.026 (0.724-1.454)	0.929
#NEUT, 10^9/L	Model 1 [b]	1.0	1.023 (0.725-1.445)	1.181 (0.843-1.654)	0.330
	Model 2 [c]	1.0	1.031 (0.729-1.457)	1.222 (0.871-1.716)	0.242
	Model 3 [d]	1.0	1.069 (0.744-1.536)	1.208 (0.844-1.729)	0.298
#LYMPH, 10^9/L	Model 1 [b]	1.0	0.549 (0.395-0.764)	0.510 (0.362-0.718)	<0.001
	Model 2 [c]	1.0	0.720 (0.516-1.005)	0.799 (0.562-1.136)	0.142
	Model 3 [d]	1.0	0.684 (0.483-0.969)	0.758 (0.524-1.095)	0.083
#MONO, 10^9/L	Model 1 [b]	1.0	0.794 (0.552-1.142)	1.406 (1.018-1.942)	0.033
	Model 2 [c]	1.0	0.779 (0.541-1.121)	1.258 (0.905-1.749)	0.151
	Model 3 [d]	1.0	0.802 (0.550-1.169)	1.217 (0.863-1.717)	0.242
#BASO, 10^9/L	Model 1 [b]	1.0	1.413 (1.002-1.993)	0.952 (0.673-1.347)	0.904
	Model 2 [c]	1.0	1.410 (0.999-1.990)	1.006 (0.711-1.423)	0.675
	Model 3 [d]	1.0	1.383 (0.964-1.986)	1.007 (0.703-1.441)	0.714
#EOS, 10^9/L	Model 1 [b]	1.0	0.896 (0.641-1.253)	0.882 (0.634-1.228)	0.446
	Model 2 [c]	1.0	0.920 (0.658-1.288)	0.884 (0.634-1.233)	0.462
	Model 3 [d]	1.0	0.948 (0.668-1.345)	0.884 (0.624-1.253)	0.489
NEUT%, %	Model 1 [b]	1.0	1.182 (0.815-1.713)	1.809 (1.286-2.546)	<0.001
	Model 2 [c]	1.0	1.070 (0.737-1.553)	1.410 (0.998-1.993)	0.041
	Model 3 [d]	1.0	1.048 (0.708-1.550)	1.454 (1.012-2.090)	0.032
LYMPH%, %	Model 1 [b]	1.0	0.541 (0.389-0.752)	0.484 (0.343-0.682)	<0.001
	Model 2 [c]	1.0	0.674 (0.483-0.941)	0.687 (0.482-0.978)	0.022
	Model 3 [d]	1.0	0.628 (0.444-0.888)	0.660 (0.456-0.958)	0.013
MONO%, %	Model 1 [b]	1.0	1.191 (0.825-1.720)	1.766 (1.260-2.474)	0.001
	Model 2 [c]	1.0	1.067 (0.738-1.545)	1.334 (0.945-1.884)	0.090
	Model 3 [d]	1.0	1.092 (0.743-1.603)	1.368 (0.950-1.970)	0.082
BASO%, %	Model 1 [b]	1.0	0.942 (0.671-1.321)	0.849 (0.601-1.201)	0.357
	Model 2 [c]	1.0	0.975 (0.695-1.368)	0.848 (0.600-1.199)	0.375
	Model 3 [d]	1.0	0.955 (0.670-1.360)	0.886 (0.619-1.268)	0.509
EOS%, %	Model 1 [b]	1.0	0.663 (0.467-0.940)	0.823 (0.597-1.134)	0.192
	Model 2 [c]	1.0	0.654 (0.461-0.928)	0.792 (0.574-1.092)	0.128
	Model 3 [d]	1.0	0.706 (0.491-1.015)	0.815 (0.582-1.143)	0.210

[a] *P* values for trend.

[b] Model 1: Crude risk.

[c] Model 2: Adjusted for age and gender.

[d] Model 3: Further adjusted for smoking status, obesity or overweight, hypertension, dyslipidemia, diabetes, stroke and family history of premature CAD.

Supplement table 7. Odds ratios (95% confidence intervals) for non-cardiovascular death according to hematologic indices levels (per 1-SD increment).

Hematologic indices	Non-cardiovascular death				
	Model	T1	T2	T3	P_{trend}[a]
RBC, 10^{12}/L	Model 1 [b]	1.0	0.324 (0.197-0.530)	0.448 (0.290-0.691)	<0.001
	Model 2 [c]	1.0	0.425 (0.256-0.706)	0.678 (0.424-1.083)	0.047
	Model 3 [d]	1.0	0.500 (0.299-0.836)	0.707 (0.431-1.160)	0.091
HGB, g/L	Model 1 [b]	1.0	0.463 (0.293-0.731)	0.516 (0.332-0.804)	0.002
	Model 2 [c]	1.0	0.549 (0.341-0.884)	0.712 (0.436-1.163)	0.117
	Model 3 [d]	1.0	0.570 (0.345-0.944)	0.749 (0.448-1.253)	0.189
HCT, %	Model 1 [b]	1.0	0.494 (0.317-0.770)	0.496 (0.315-0.780)	0.001
	Model 2 [c]	1.0	0.608 (0.385-0.960)	0.680 (0.417-1.109)	0.083
	Model 3 [d]	1.0	0.609 (0.374-0.990)	0.701 (0.419-1.173)	0.118
MCV, fL	Model 1 [b]	1.0	0.915 (0.570-1.469)	1.282 (0.830-1.981)	0.254
	Model 2 [c]	1.0	0.809 (0.503-1.299)	0.858 (0.551-1.336)	0.527
	Model 3 [d]	1.0	0.878 (0.537-1.435)	0.865 (0.540-1.387)	0.561
MCH, pg	Model 1 [b]	1.0	0.969 (0.620-1.515)	0.999 (0.637-1.567)	0.994
	Model 2 [c]	1.0	0.890 (0.567-1.394)	0.744 (0.470-1.179)	0.207
	Model 3 [d]	1.0	0.852 (0.534-1.359)	0.732 (0.454-1.181)	0.201
MCHC, g/L	Model 1 [b]	1.0	0.618 (0.395-0.968)	0.729 (0.469-1.133)	0.126
	Model 2 [c]	1.0	0.713 (0.452-1.124)	0.892 (0.563-1.414)	0.541
	Model 3 [d]	1.0	0.716 (0.447-1.147)	0.827 (0.510-1.341)	0.379
RDW-CV, %	Model 1 [b]	1.0	1.287 (0.753-2.200)	2.580 (1.593-4.180)	<0.001
	Model 2 [c]	1.0	0.984 (0.573-1.687)	1.612 (0.984-2.640)	0.027
	Model 3 [d]	1.0	1.189 (0.658-2.146)	1.863 (1.078-3.217)	0.012
RDW-SD, fL	Model 1 [b]	1.0	0.699 (0.394-1.241)	2.465 (1.583-3.839)	<0.001
	Model 2 [c]	1.0	0.512 (0.287-0.914)	1.308 (0.821-2.083)	0.069
	Model 3 [d]	1.0	0.489 (0.262-0.912)	1.308 (0.797-2.148)	0.078
PLT, 10^9/L	Model 1 [b]	1.0	0.385 (0.240-0.618)	0.470 (0.302-0.732)	<0.001
	Model 2 [c]	1.0	0.476 (0.296-0.767)	0.726 (0.462-1.142)	0.070
	Model 3 [d]	1.0	0.455 (0.274-0.756)	0.729 (0.459-1.157)	0.082
PCT, %	Model 1 [b]	1.0	0.388 (0.233-0.645)	0.525 (0.336-0.821)	0.001
	Model 2 [c]	1.0	0.525 (0.314-0.879)	0.800 (0.506-1.265)	0.171
	Model 3 [d]	1.0	0.439 (0.249-0.775)	0.773 (0.485-1.231)	0.118
MPV, fL	Model 1 [b]	1.0	1.231 (0.780-1.943)	1.367 (0.868-2.152)	0.176
	Model 2 [c]	1.0	1.183 (0.749-1.868)	1.288 (0.817-2.030)	0.275
	Model 3 [d]	1.0	1.237 (0.774-1.977)	1.259 (0.782-2.026)	0.338
PDW, %	Model 1 [b]	1.0	1.031 (0.650-1.637)	1.189 (0.760-1.861)	0.446
	Model 2 [c]	1.0	1.064 (0.670-1.689)	1.147 (0.732-1.797)	0.550
	Model 3 [d]	1.0	1.168 (0.729-1.874)	1.109 (0.689-1.786)	0.668

P-LCR, %	Model 1 [b]	1.0	1.447 (0.908-2.306)	1.409 (0.877-2.262)	0.164
	Model 2 [c]	1.0	1.415 (0.888-2.257)	1.304 (0.812-2.097)	0.292
	Model 3 [d]	1.0	1.440 (0.893-2.322)	1.266 (0.771-2.078)	0.364
WBC, 10^9/L	Model 1 [b]	1.0	0.698 (0.452-1.079)	0.651 (0.414-1.023)	0.054
	Model 2 [c]	1.0	0.752 (0.486-1.164)	0.772 (0.491-1.214)	0.231
	Model 3 [d]	1.0	0.827 (0.524-1.305)	0.782 (0.484-1.263)	0.300
#NEUT, 10^9/L	Model 1 [b]	1.0	0.977 (0.630-1.515)	0.866 (0.548-1.369)	0.542
	Model 2 [c]	1.0	0.971 (0.626-1.507)	0.883 (0.558-1.397)	0.599
	Model 3 [d]	1.0	0.999 (0.631-1.580)	0.868 (0.536-1.408)	0.573
#LYMPH, 10^9/L	Model 1 [b]	1.0	0.554 (0.360-0.852)	0.436 (0.272-0.699)	<0.001
	Model 2 [c]	1.0	0.772 (0.500-1.192)	0.759 (0.467-1.232)	0.858
	Model 3 [d]	1.0	0.720 (0.456-1.136)	0.701 (0.421-1.167)	0.122
#MONO, 10^9/L	Model 1 [b]	1.0	0.825 (0.513-1.326)	1.323 (0.858-2.038)	0.199
	Model 2 [c]	1.0	0.801 (0.498-1.289)	1.109 (0.714-1.723)	0.620
	Model 3 [d]	1.0	0.796 (0.488-1.299)	0.999 (0.633-1.579)	0.987
#BASO, 10^9/L	Model 1 [b]	1.0	1.157 (0.721-1.855)	0.770 (0.476-1.246)	0.391
	Model 2 [c]	1.0	1.139 (0.709-1.828)	0.812 (0.502-1.314)	0.506
	Model 3 [d]	1.0	1.085 (0.660-1.783)	0.785 (0.478-1.291)	0.415
#EOS, 10^9/L	Model 1 [b]	1.0	0.803 (0.515-1.254)	0.774 (0.498-1.203)	0.239
	Model 2 [c]	1.0	0.824 (0.527-1.288)	0.757 (0.486-1.179)	0.209
	Model 3 [d]	1.0	0.808 (0.506-1.291)	0.748 (0.472-1.187)	0.209
NEUT%, %	Model 1 [b]	1.0	0.957 (0.586-1.563)	1.583 (1.020-2.457)	0.033
	Model 2 [c]	1.0	0.836 (0.512-1.368)	1.145 (0.734-1.788)	0.474
	Model 3 [d]	1.0	0.849 (0.506-1.424)	1.223 (0.766-1.955)	0.322
LYMPH%, %	Model 1 [b]	1.0	0.504 (0.322-0.791)	0.525 (0.337-0.820)	0.003
	Model 2 [c]	1.0	0.667 (0.424-1.050)	0.838 (0.530-1.326)	0.329
	Model 3 [d]	1.0	0.625 (0.392-0.997)	0.789 (0.485-1.284)	0.211
MONO%, %	Model 1 [b]	1.0	1.193 (0.736-1.934)	1.682 (1.075-2.633)	0.021
	Model 2 [c]	1.0	1.024 (0.630-1.664)	1.162 (0.735-1.837)	0.503
	Model 3 [d]	1.0	0.984 (0.596-1.625)	1.101 (0.680-1.782)	0.671
BASO%, %	Model 1 [b]	1.0	0.847 (0.537-1.336)	0.764 (0.477-1.223)	0.236
	Model 2 [c]	1.0	0.877 (0.556-1.384)	0.759 (0.474-1.215)	0.239
	Model 3 [d]	1.0	0.804 (0.498-1.298)	0.761 (0.468-1.238)	0.232
EOS%, %	Model 1 [b]	1.0	0.645 (0.400-1.039)	0.929 (0.610-1.413)	0.675
	Model 2 [c]	1.0	0.627 (0.389-1.011)	0.875 (0.574-1.332)	0.496
	Model 3 [d]	1.0	0.636 (0.385-1.049)	0.910 (0.587-1.413)	0.646

[a] *P* values for trend.

[b] Model 1: Crude risk.

[c] Model 2: Adjusted for age and gender.

[d] Model 3: Further adjusted for smoking status, obesity or overweight, hypertension, dyslipidemia, diabetes, stroke and family history of premature CAD.

Supplement table 8. Odds ratios (95% confidence intervals) for cardiovascular death according to hematologic indices levels (per 1-SD increment).

Hematologic indices	Cardiovascular death				
	Model	T1	T2	T3	P_{trend}[a]
RBC, 10^{12}/L	Model 1 [b]	1.0	0.494 (0.292-0.834)	0.578 (0.352-0.948)	0.021
	Model 2 [c]	1.0	0.632 (0.368-1.086)	0.857 (0.497-1.479)	0.481
	Model 3 [d]	1.0	0.754 (0.432-1.317)	0.989 (0.561-1.746)	0.898
HGB, g/L	Model 1 [b]	1.0	0.510 (0.301-0.863)	0.637 (0.390-1.042)	0.053
	Model 2 [c]	1.0	0.626 (0.361-1.085)	0.932 (0.527-1.648)	0.689
	Model 3 [d]	1.0	0.774 (0.438-1.366)	1.118 (0.617-2.026)	0.781
HCT, %	Model 1 [b]	1.0	0.580 (0.349-0.964)	0.650 (0.394-1.074)	0.074
	Model 2 [c]	1.0	0.718 (0.424-1.216)	0.936 (0.535-1.637)	0.723
	Model 3 [d]	1.0	0.896 (0.517-1.553)	1.129 (0.626-2.036)	0.724
MCV, fL	Model 1 [b]	1.0	1.438 (0.858-2.410)	1.205 (0.706-2.057)	0.506
	Model 2 [c]	1.0	1.345 (0.802-2.256)	0.948 (0.551-1.630)	0.798
	Model 3 [d]	1.0	1.301 (0.759-2.230)	0.952 (0.539-1.682)	0.835
MCH, pg	Model 1 [b]	1.0	0.962 (0.573-1.618)	1.128 (0.680-1.871)	0.641
	Model 2 [c]	1.0	0.943 (0.560-1.589)	1.011 (0.602-1.699)	0.964
	Model 3 [d]	1.0	0.936 (0.541-1.619)	1.065 (0.620-1.828)	0.817
MCHC, g/L	Model 1 [b]	1.0	0.882 (0.549-1.415)	0.582 (0.335-1.012)	0.06
	Model 2 [c]	1.0	1.010 (0.624-1.636)	0.714 (0.401-1.272)	0.29
	Model 3 [d]	1.0	1.001 (0.603-1.664)	0.757 (0.420-1.366)	0.386
RDW-CV, %	Model 1 [b]	1.0	1.351 (0.751-2.429)	2.312 (1.345-3.974)	0.002
	Model 2 [c]	1.0	1.141 (0.632-2.061)	1.702 (0.976-2.969)	0.044
	Model 3 [d]	1.0	1.178 (0.642-2.161)	1.608 (0.899-2.875)	0.093
RDW-SD, fL	Model 1 [b]	1.0	1.363 (0.754-2.463)	2.332 (1.357-4.010)	0.001
	Model 2 [c]	1.0	1.132 (0.623-2.056)	1.595 (0.906-2.806)	0.084
	Model 3 [d]	1.0	1.233 (0.671-2.266)	1.525 (0.838-2.775)	0.16
PLT, 10^9/L	Model 1 [b]	1.0	0.616 (0.377-1.004)	0.472 (0.277-0.803)	0.004
	Model 2 [c]	1.0	0.680 (0.415-1.114)	0.587 (0.339-1.015)	0.044
	Model 3 [d]	1.0	0.710 (0.426-1.183)	0.563 (0.317-1.001)	0.043
PCT, %	Model 1 [b]	1.0	0.635 (0.379-1.064)	0.594 (0.355-0.995)	0.034
	Model 2 [c]	1.0	0.746 (0.442-1.260)	0.740 (0.434-1.260)	0.226
	Model 3 [d]	1.0	0.724 (0.419-1.253)	0.690 (0.396-1.201)	0.158
MPV, fL	Model 1 [b]	1.0	1.529 (0.872-2.680)	2.161 (1.268-3.682)	0.004
	Model 2 [c]	1.0	1.468 (0.837-2.575)	2.014 (1.181-3.438)	0.009
	Model 3 [d]	1.0	1.426 (0.795-2.560)	2.058 (1.185-3.572)	0.009
PDW, %	Model 1 [b]	1.0	1.576 (0.895-2.774)	2.035 (1.184-3.498)	0.01
	Model 2 [c]	1.0	1.587 (0.901-2.794)	1.932 (1.123-3.324)	0.018
	Model 3 [d]	1.0	1.514 (0.841-2.727)	1.969 (1.124-3.451)	0.017
P-LCR, %	Model 1 [b]	1.0	1.664 (0.940-2.945)	2.076 (1.194-3.610)	0.01

	Model 2 [c]	1.0	1.614 (0.912-2.858)	1.911 (1.098-3.328)	0.023
	Model 3 [d]	1.0	1.573 (0.869-2.848)	1.967 (1.108-3.492)	0.021
WBC, 10^9/L	Model 1 [b]	1.0	0.706 (0.407-1.223)	1.212 (0.746-1.967)	0.409
	Model 2 [c]	1.0	0.756 (0.436-1.312)	1.407 (0.864-2.292)	0.165
	Model 3 [d]	1.0	0.824 (0.464-1.463)	1.425 (0.846-2.402)	0.177
#NEUT, 10^9/L	Model 1 [b]	1.0	1.107 (0.632-1.940)	1.727 (1.032-2.891)	0.031
	Model 2 [c]	1.0	1.143 (0.651-2.007)	1.846 (1.098-3.102)	0.017
	Model 3 [d]	1.0	1.209 (0.669-2.184)	1.850 (1.066-3.213)	0.025
#LYMPH, 10^9/L	Model 1 [b]	1.0	0.544 (0.326-0.909)	0.616 (0.374-1.015)	0.044
	Model 2 [c]	1.0	0.663 (0.395-1.113)	0.847 (0.506-1.417)	0.436
	Model 3 [d]	1.0	0.652 (0.379-1.120)	0.834 (0.487-1.429)	0.421
#MONO, 10^9/L	Model 1 [b]	1.0	0.749 (0.426-1.320)	1.509 (0.929-2.452)	0.081
	Model 2 [c]	1.0	0.751 (0.426-1.324)	1.478 (0.897-2.433)	0.107
	Model 3 [d]	1.0	0.819 (0.453-1.480)	1.568 (0.926-2.656)	0.081
#BASO, 10^9/L	Model 1 [b]	1.0	1.817 (1.093-3.021)	1.237 (0.744-2.057)	0.255
	Model 2 [c]	1.0	1.837 (1.104-3.057)	1.308 (0.786-2.176)	0.175
	Model 3 [d]	1.0	1.858 (1.088-3.173)	1.357 (0.801-2.299)	0.155
#EOS, 10^9/L	Model 1 [b]	1.0	1.038 (0.622-1.732)	1.044 (0.632-1.725)	0.865
	Model 2 [c]	1.0	1.071 (0.641-1.789)	1.077 (0.649-1.785)	0.771
	Model 3 [d]	1.0	1.178 (0.692-2.006)	1.100 (0.644-1.881)	0.712
NEUT%, %	Model 1 [b]	1.0	1.576 (0.884-2.811)	2.199 (1.274-3.797)	0.004
	Model 2 [c]	1.0	1.495 (0.837-2.671)	1.887 (1.086-3.277)	0.024
	Model 3 [d]	1.0	1.379 (0.754-2.523)	1.830 (1.032-3.246)	0.036
LYMPH%, %	Model 1 [b]	1.0	0.589 (0.362-0.958)	0.432 (0.252-0.741)	0.001
	Model 2 [c]	1.0	0.679 (0.415-1.110)	0.530 (0.304-0.922)	0.019
	Model 3 [d]	1.0	0.635 (0.378-1.064)	0.537 (0.302-0.954)	0.024
MONO%, %	Model 1 [b]	1.0	1.190 (0.675-2.096)	1.870 (1.120-3.123)	0.014
	Model 2 [c]	1.0	1.129 (0.639-1.995)	1.587 (0.939-2.685)	0.074
	Model 3 [d]	1.0	1.241 (0.682-2.260)	1.780 (1.017-3.117)	0.038
BASO%, %	Model 1 [b]	1.0	1.076 (0.649-1.786)	0.972 (0.581-1.624)	0.958
	Model 2 [c]	1.0	1.109 (0.668-1.841)	0.975 (0.583-1.631)	0.984
	Model 3 [d]	1.0	1.178 (0.694-1.998)	1.063 (0.623-1.813)	0.758
EOS%, %	Model 1 [b]	1.0	0.686 (0.410-1.145)	0.695 (0.422-1.145)	0.133
	Model 2 [c]	1.0	0.688 (0.412-1.149)	0.686 (0.416-1.133)	0.123
	Model 3 [d]	1.0	0.794 (0.469-1.345)	0.694 (0.407-1.185)	0.173

[a] *P* values for trend.

[b] Model 1: Crude risk.

[c] Model 2: Adjusted for age and gender.

[d] Model 3: Further adjusted for smoking status, obesity or overweight, hypertension, dyslipidemia, diabetes, stroke and family history of premature CAD.
